# Supplementary figures and images for: Machine learning with random subspace ensembles identifies antimicrobial resistance determinants from pan-genomes of three pathogens
Source: PLoS Comput Biol. 2020 Mar 2;16(3):e1007608. doi: 10.1371/journal.pcbi.1007608 (PMC7067475; doi:10.1371/journal.pcbi.1007608)

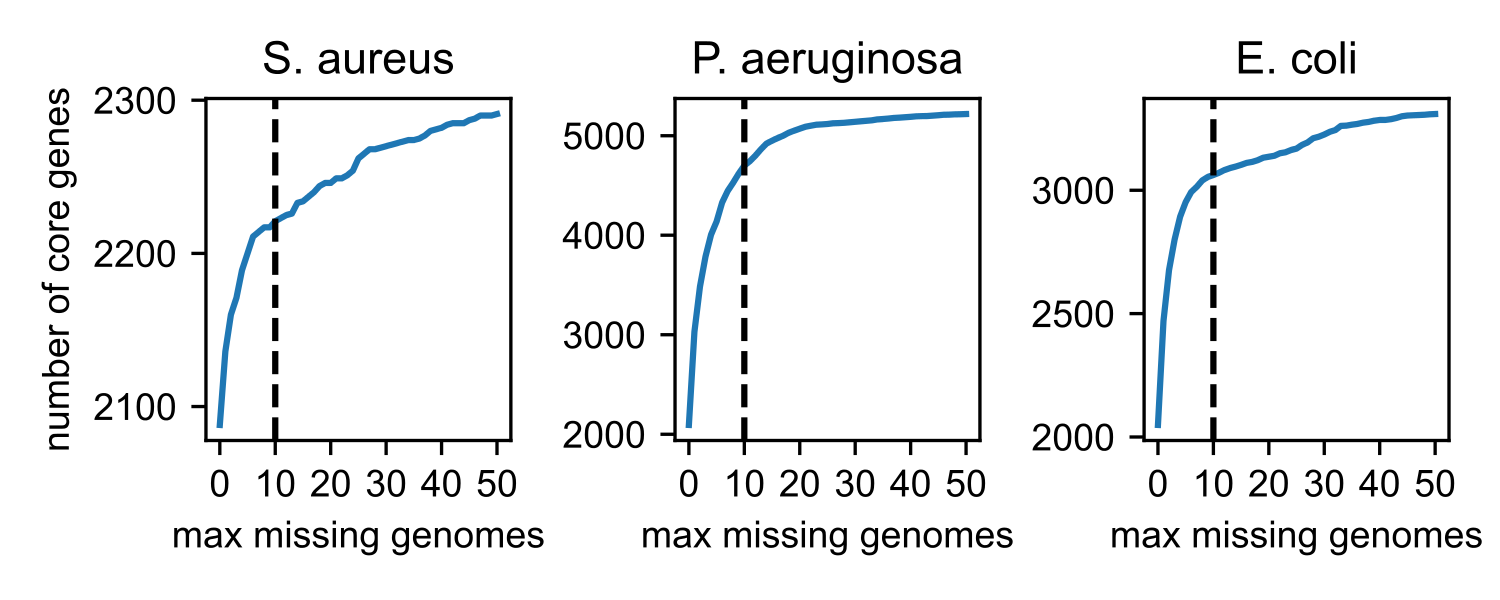

Supplement: S1 Fig — For each pan-genome, the threshold for classifying a gene as a core gene was relaxed from allowing at most 0 to at most 50 genomes to be missing the gene. The threshold of 10 genomes used for subsequent analyses is shown. (TIF) [file pcbi.1007608.s001.tif]

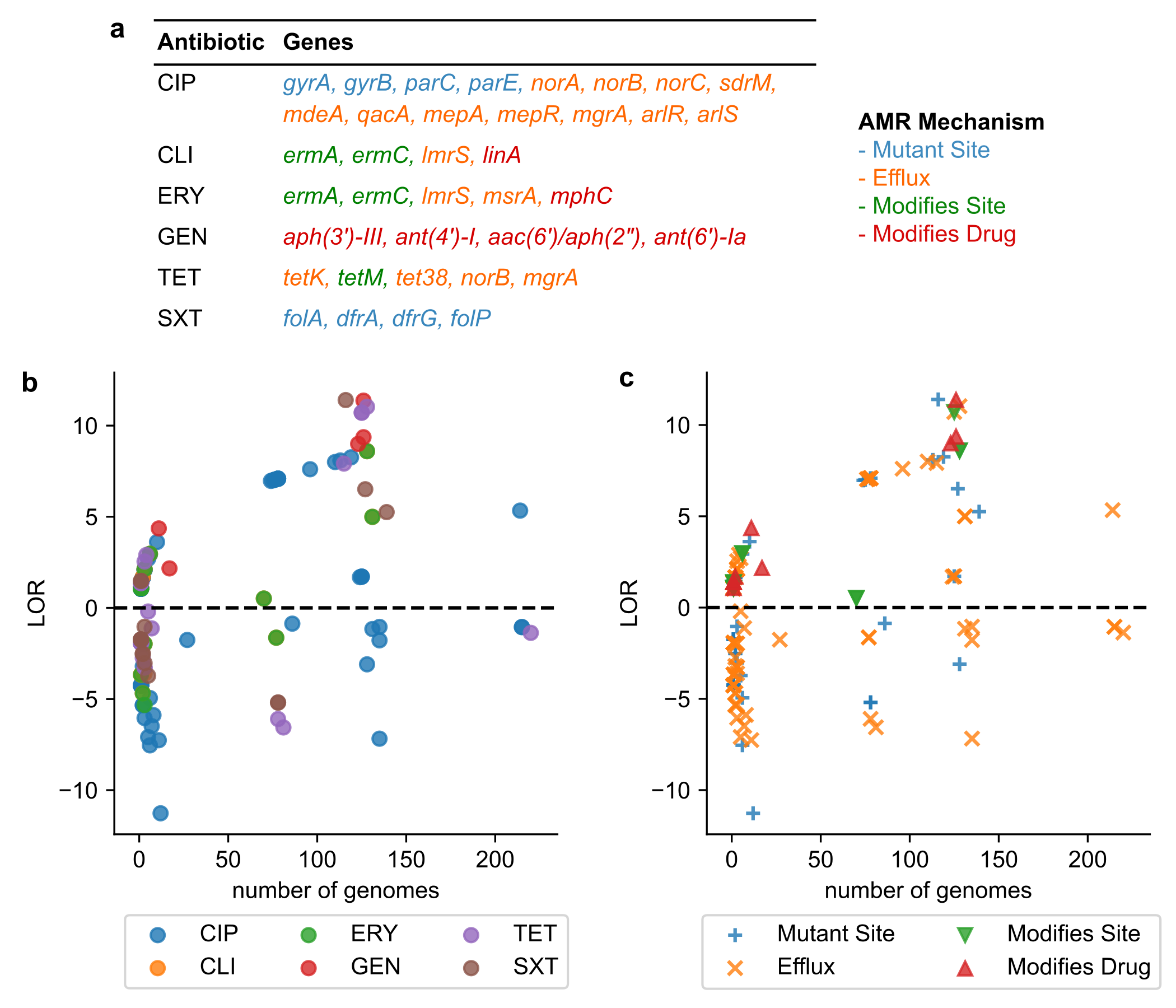

Supplement: S2 Fig — (a) Each known AMR gene detected in the S. aureus pan-genome was assigned to one of four broad mechanistic categories. For each allele of each known AMR gene, the number of genomes it is present in and the log2 odds ratio (LOR) for resistance against the appropriate drug was plotted, labeled by (b) drug or (c) mechanism. (TIF) [file pcbi.1007608.s002.tif]

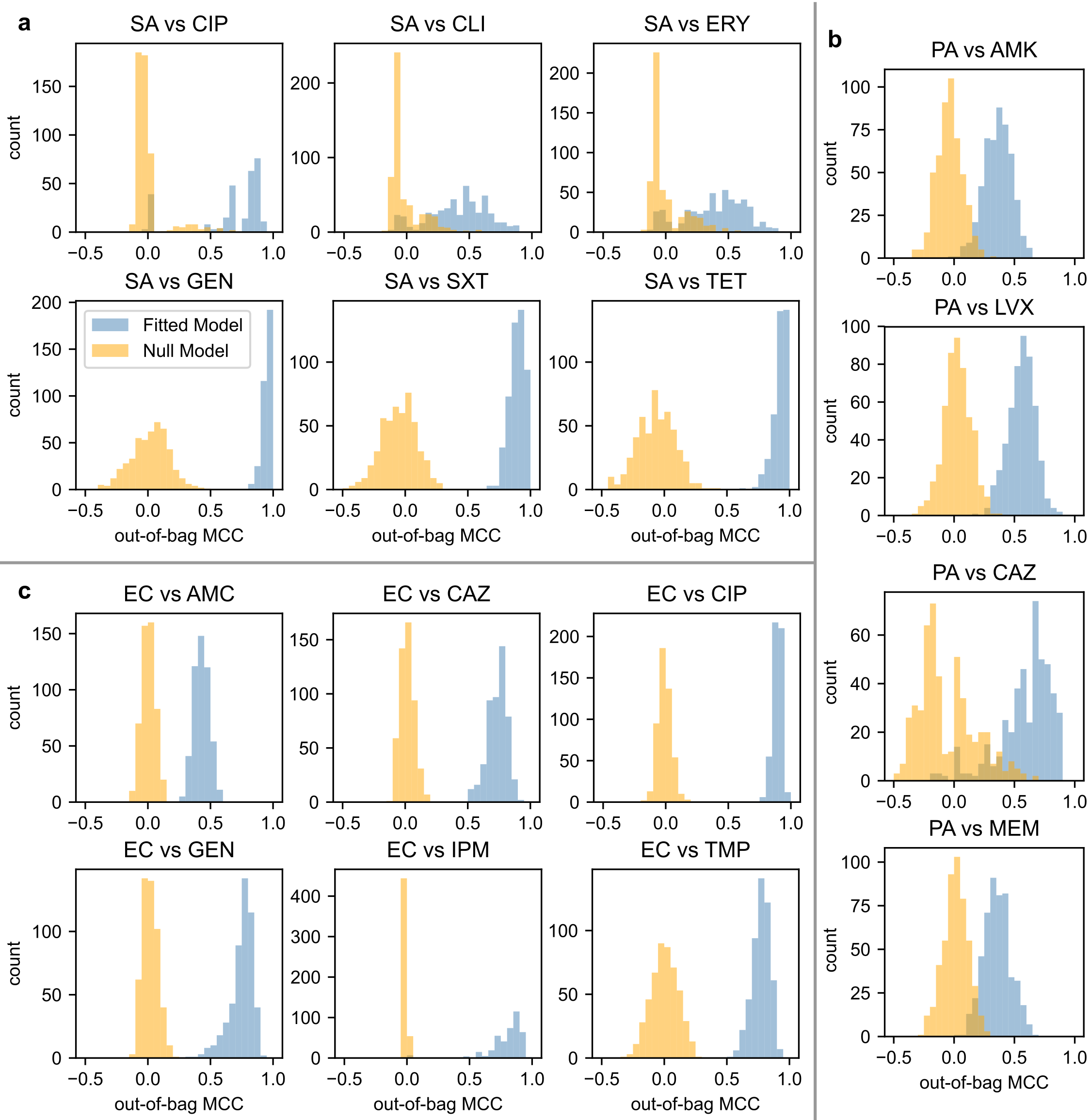

Supplement: S3 Fig — For each of the 16 organism-antibiotic cases across (a) S. aureus, (b) P. aeruginosa, and (c) E. coli, the performance of each of the 500 constituent SVMs used in the corresponding SVM-RSE was assessed as the Matthew’s correlation coefficients (MCCs) when predicting AMR phenotypes for out-of-bag genomes (those not used for training), shown in blue. The out-of-bag MCCs of constituent SVMs of SVM-RSEs trained using randomly shuffled AMR phenotype annotations are shown in orange. (TIF) [file pcbi.1007608.s003.tif]

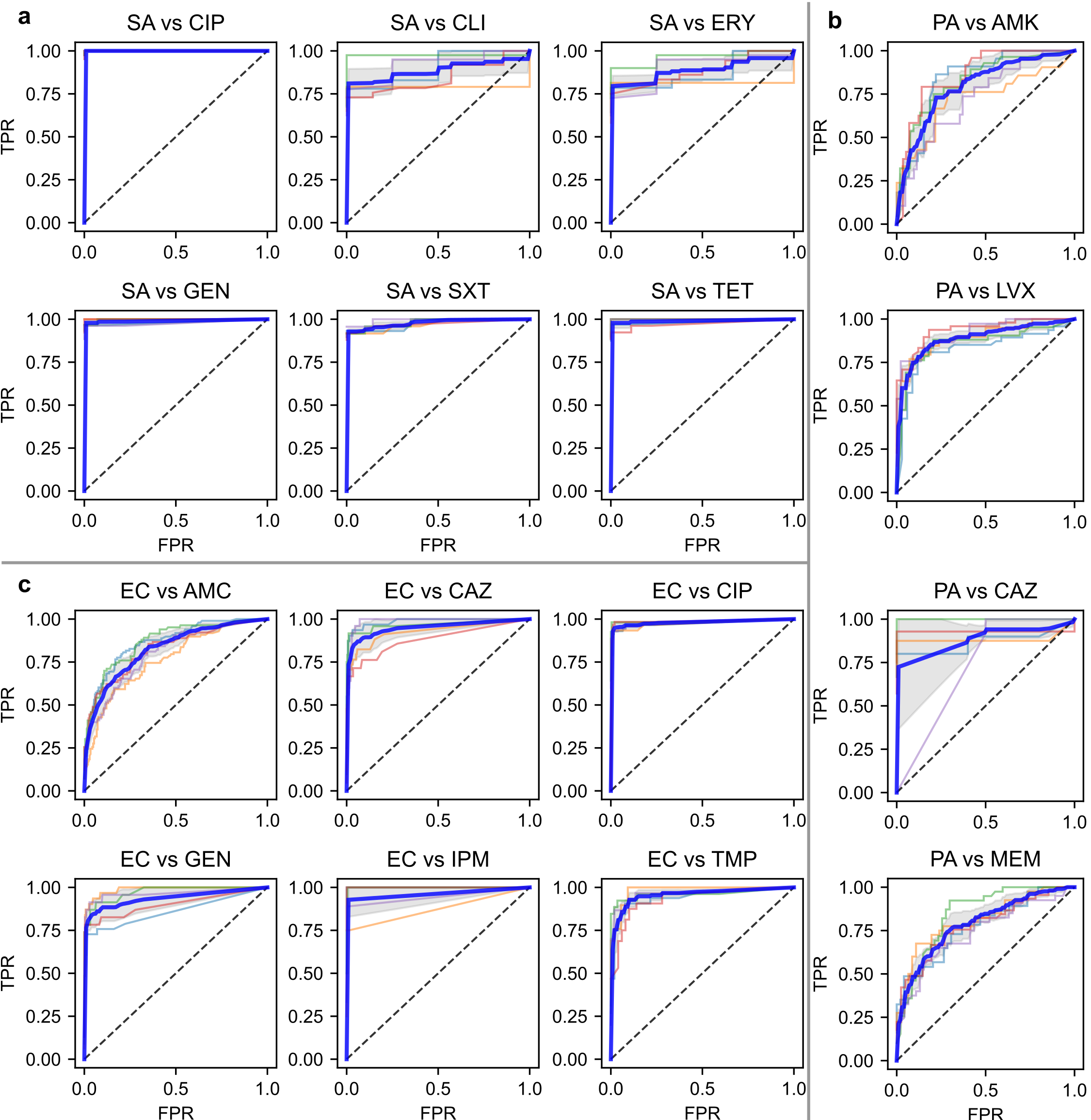

Supplement: S4 Fig — ROC curves for each of the 16 organism-antibiotic cases across (a) S. aureus, (b) P. aeruginosa, and (c) E. coli. The dark blue curves are mean ROC curves from 5-fold cross validation, the lighter curves are individual ROC curves corresponding to each fold, and the grayed areas are within one standard deviation of the mean ROC curve. (TIF) [file pcbi.1007608.s004.tif]

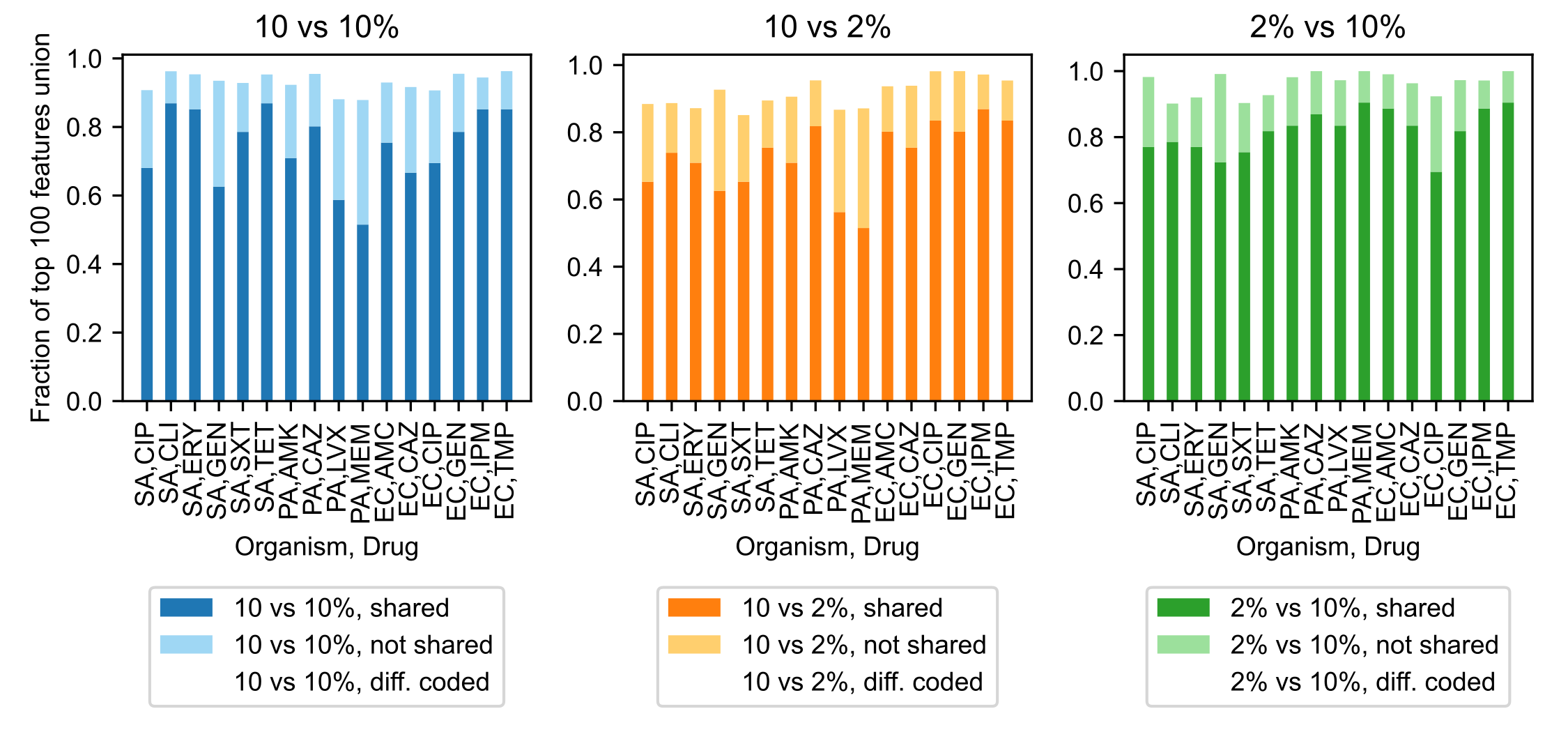

Supplement: S5 Fig — The top 100 features (top 50 resistance-associated + top 50 susceptibility-associated) were identified using SVM-RSE for three different core gene thresholds (10: missing from at most 10 genomes, 10%: missing from at most 10% of all genomes, 2%: missing from at most 2% of all genomes). For each pair of thresholds, the fraction of shared vs. non-shared features in the union of their top 100 feature sets were computed. Non-shared features were classified as either “not shared”, where both representations contain the feature, or “diff. coded”, where the feature is only available under one of the thresholds. (TIF) [file pcbi.1007608.s005.tif]

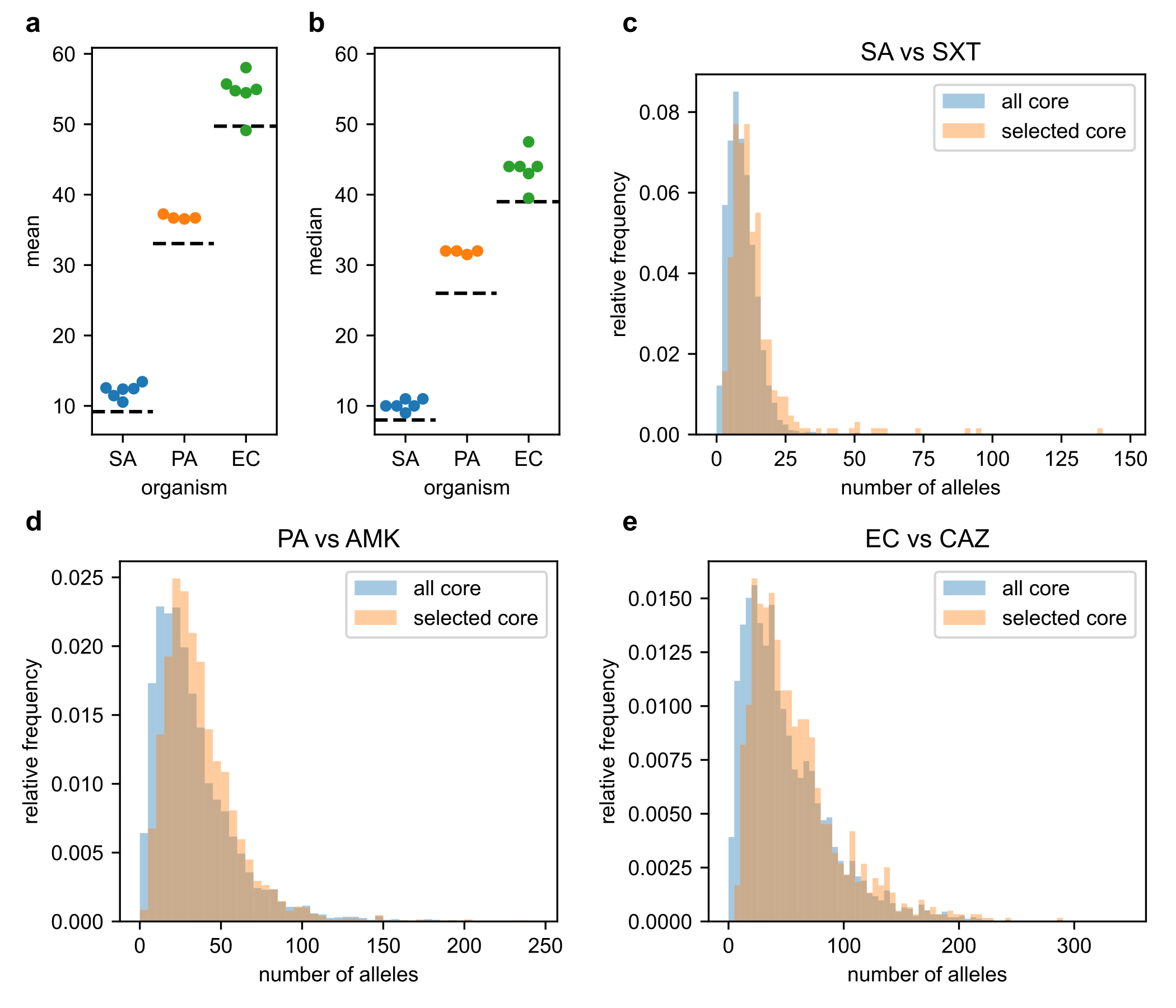

Supplement: S6 Fig — For each organism-antibiotic case, the distribution of the number of alleles of all core genes was compared to that of core genes for which at least one allele was selected by SVM-RSE to be associated with resistance or susceptibility. The (a) mean and (b) median of the selected core gene allele count is shown for each case, compared to the mean and median for all core genes of the corresponding species (dotted lines). For each species, the allele count distributions are shown for the case with the largest difference in mean allele count, (c) S. aureus vs. sulfamethoxazole/trimethoprim, (d) P. aeruginosa vs. amikacin, and (e) E. coli vs. ceftazidime. (TIF) [file pcbi.1007608.s006.tif]

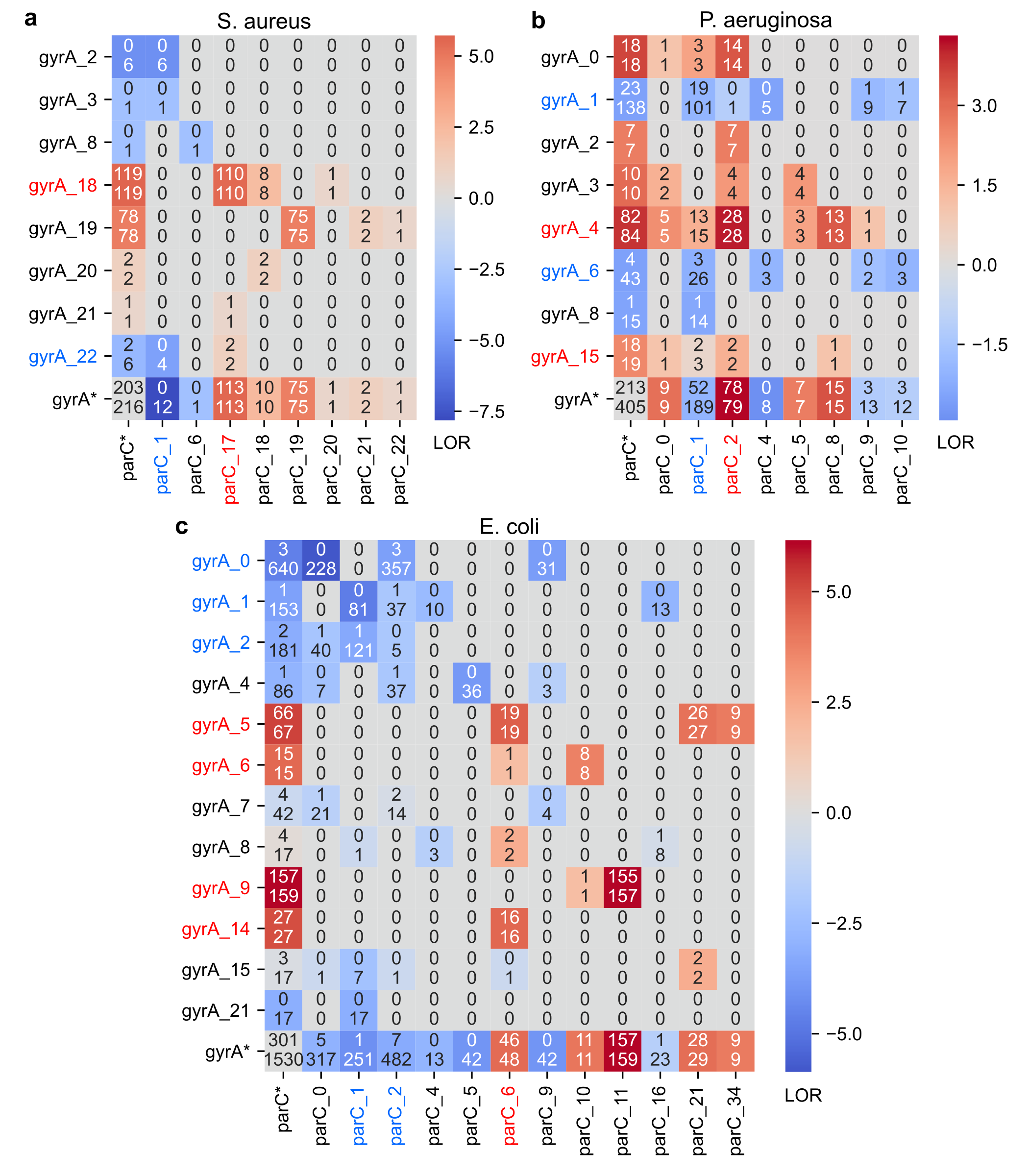

Supplement: S7 Fig — Log2 odds ratios (LORs) for fluoroquinolone resistance were calculated for each gyrA/parC allele pairing and compared to individual alleles in (a) S. aureus, (b) P. aeruginosa, and (c) E. coli. Each cell shows the number of resistant genomes with the allele above, the total number of genomes with the allele below, and is colored by LOR; row and column totals do not add up as only the top 8 (for S. aureus and P. aeruginosa) or top 12 (for E. coli) most frequently observed gyrA and parC alleles are shown. Alleles among the top 10 features detected by SVM-RSE to be associated with fluoroquinolone resistance are in red, while those the SVM-RSE associated with susceptibility are in blue. (TIF) [file pcbi.1007608.s007.tif]

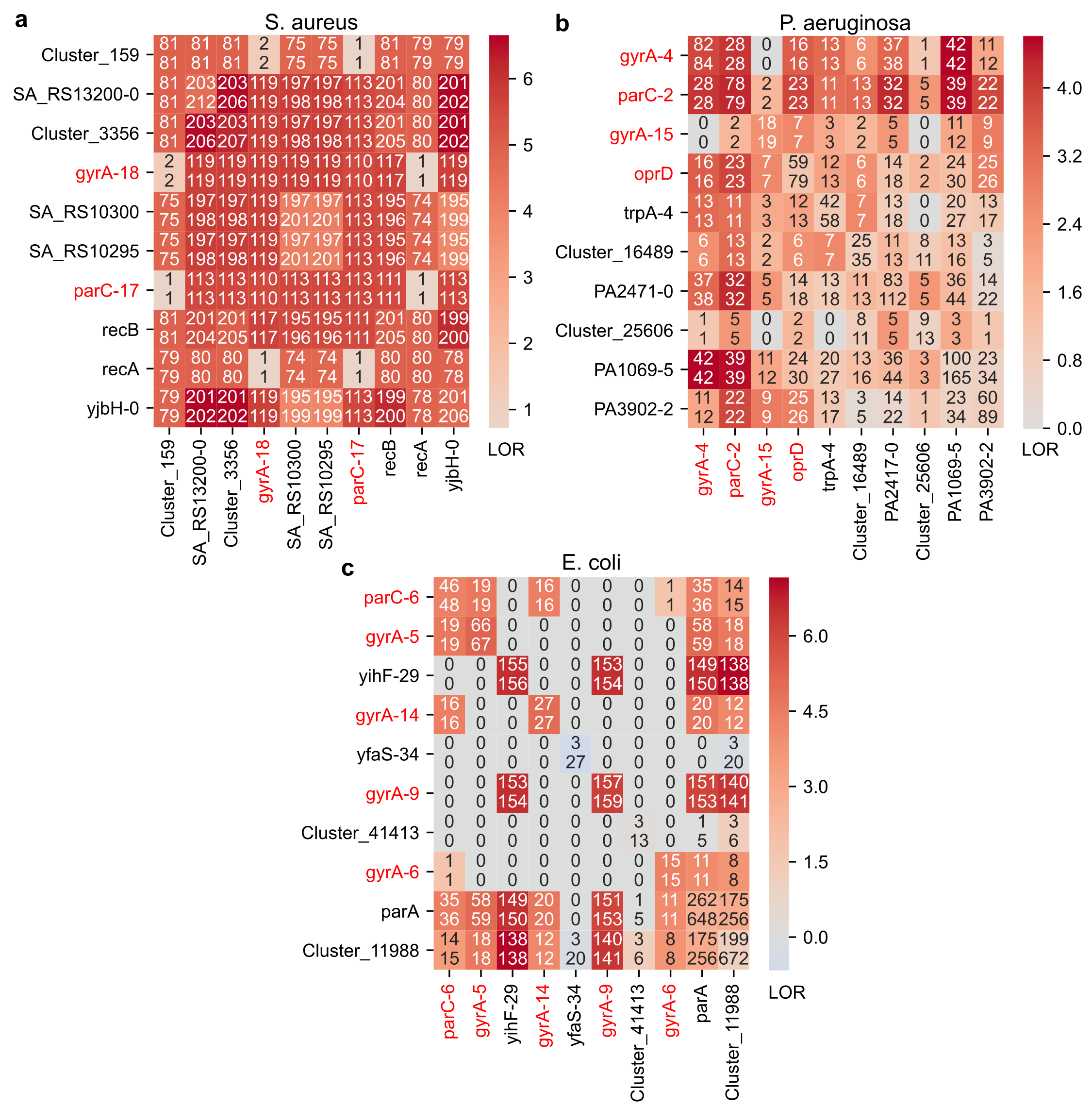

Supplement: S8 Fig — For each of the top 10 genetic features predicted by SVM-RSE to be associated with fluoroquinolone resistance in (a) S. aureus, (b) P. aeruginosa, and (c) E. coli, log2 odds ratios (LORs) for resistance were computed for each feature individually as well as for every top feature pairing. Each cell shows the number of resistant genomes with the allele above, the total number of genomes with the allele below, and is colored by LOR. Gene features are denoted by either their gene name, reference genome locus tag, or “Cluster_#” in cases the coding sequence could not be confidently mapped to a known gene. Allele features are denoted as "gene name-allele number”. Features known to confer resistance are in red. (TIF) [file pcbi.1007608.s008.tif]

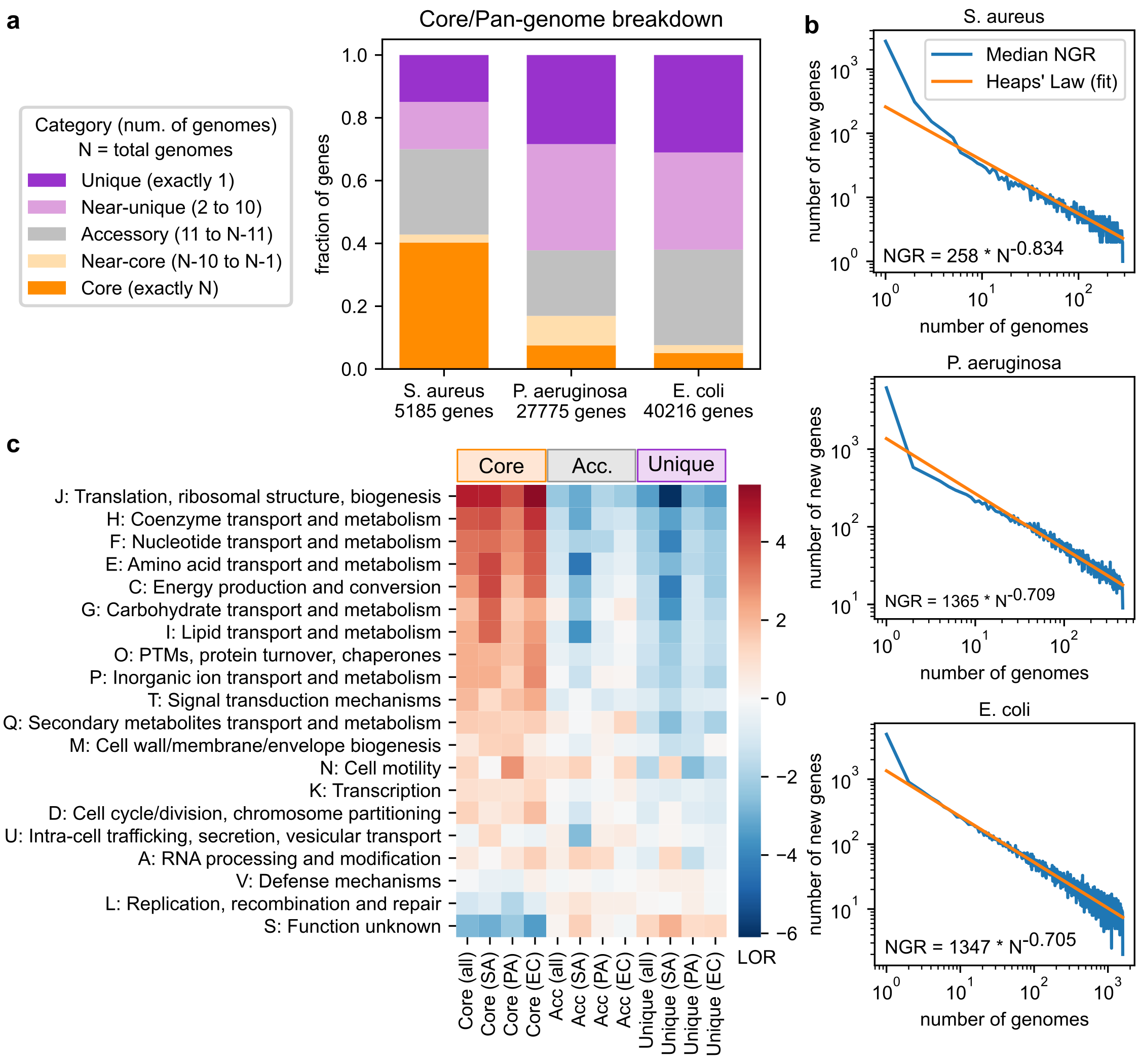

Supplement: S9 Fig — (a) Distribution of genes categorized by frequency within each pan-genome: i) core: present in all genomes, ii) near-core: missing from at most 10 genomes, iii) accessory: missing from >10 genomes and present in >10 genomes, iv) near-unique: present in 2–10 genomes, v) unique: present in exactly 1 genome. (b) Estimation of pan-genome openness using Heap’s Law. The total number of genes (pan-genome size) and number of genes in all genomes (core genome size) was computed as genomes were introduced sequentially from either the S. aureus (SA), P. aeruginosa (PA), or E. coli (EC) pan-genome. Each value represents the median from 2000 random permutations of genome order. The new gene rate (NGR) was fitted to Heap’s Law, in which a more negative exponent represents a more closed pan-genome. (c) Log2 odds ratios (LORs) between individual functional categories and the core, accessory (acc), and unique genomes for each organism individually and combined. (TIF) [file pcbi.1007608.s009.tif]

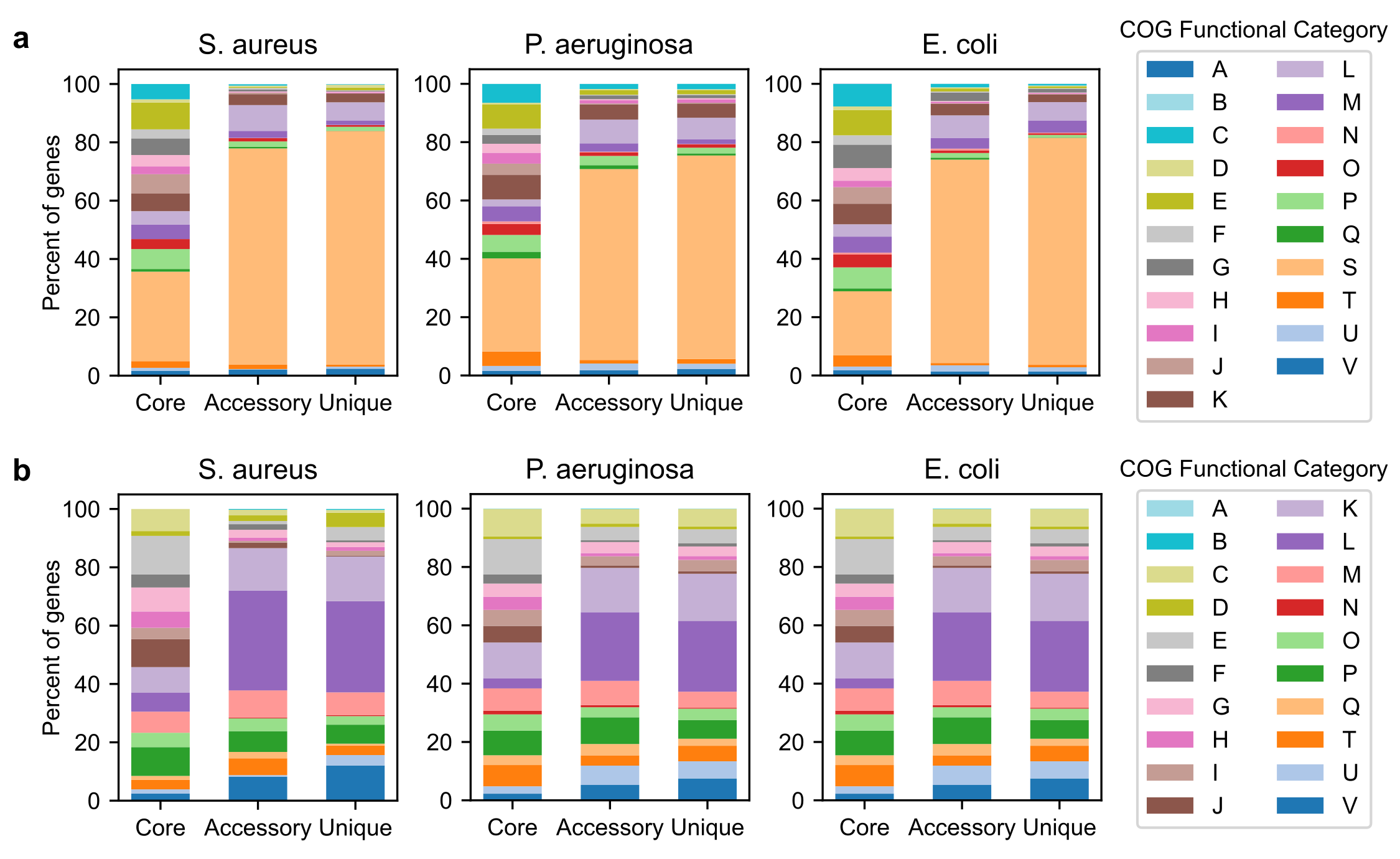

Supplement: S10 Fig — The distribution of gene functional categories based on Clusters of Orthologous Groups (COGs) in the core, accessory, and unique genomes are shown, either (a) including, or (b) excluding the “S: Function unknown” category. (TIF) [file pcbi.1007608.s010.tif]

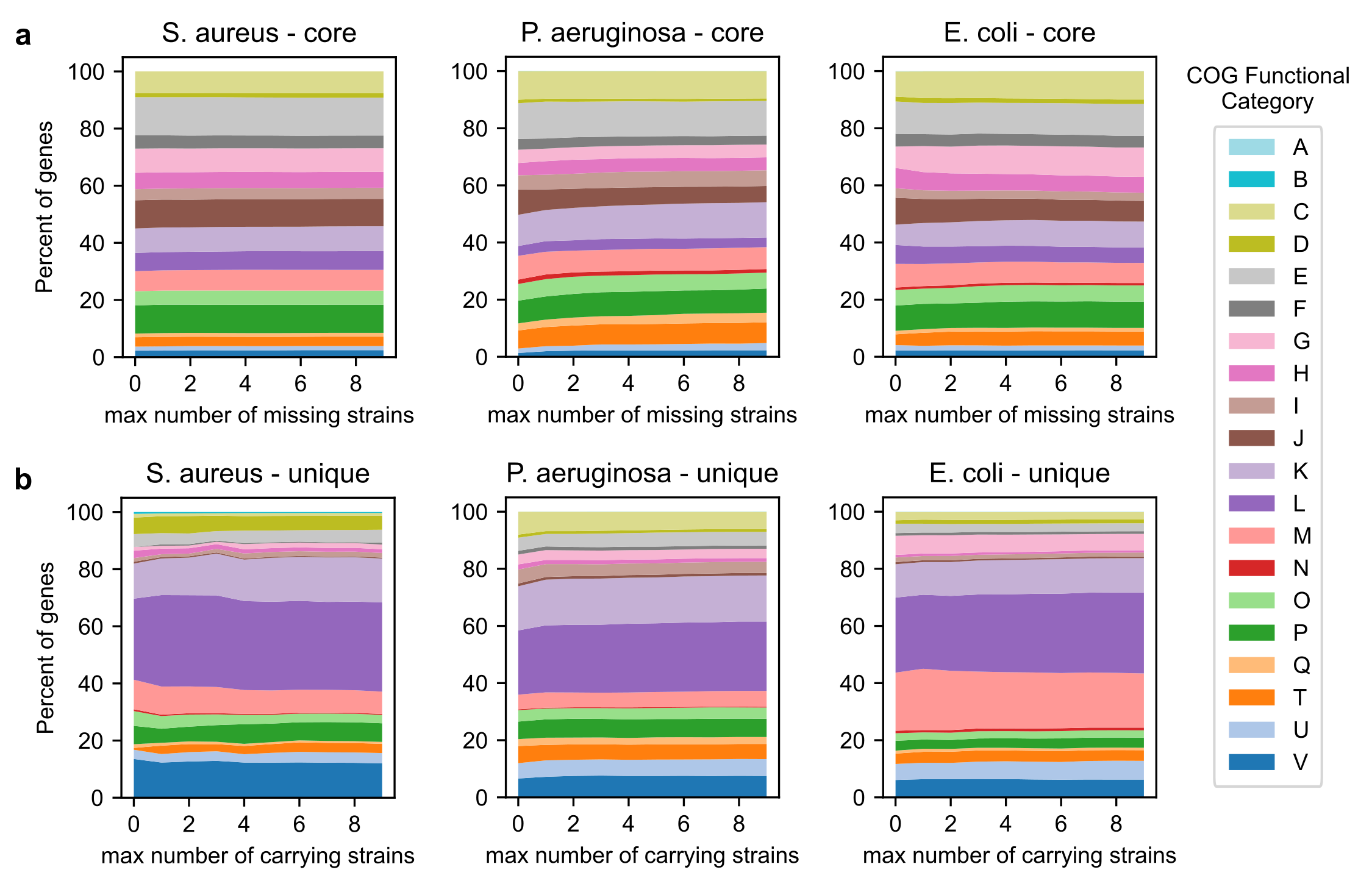

Supplement: S11 Fig — For each organism, the set of genes in the (a) core genome was assembled for different core gene thresholds (the maximum number of genomes allowed to be missing a core gene), and (b) analogously for unique genes comprising the unique genome (the maximum number of genomes allowed to carry a unique gene). The “S: Function unknown” functional category is not shown. (TIF) [file pcbi.1007608.s011.tif]
