# Supplementary material for: Machine learning with random subspace ensembles identifies antimicrobial resistance determinants from pan-genomes of three pathogens
Source: PLoS Comput Biol. 2020 Mar 2;16(3):e1007608. doi: 10.1371/journal.pcbi.1007608 (PMC7067475; doi:10.1371/journal.pcbi.1007608)
Supplement: S1 Table — (DOCX) [file pcbi.1007608.s012.docx]

| **S1 Table. Number of core, accessory, and unique genes and alleles in the pan-genome of each organism.** | | | |
| --- | --- | --- | --- |
|  | ***S. aureus*** | ***P. aeruginosa*** | ***E. coli*** |
| core genes | 2221 | 4700 | 3062 |
| accessory genes | 1409 | 5795 | 12218 |
| unique genes | 1555 | 17280 | 24936 |
| **total genes** | **5185** | **27775** | **40216** |
| core gene alleles | 20390 | 155350 | 152264 |
| accessory gene alleles | 9083 | 89151 | 228449 |
| unique gene alleles | 2262 | 27304 | 39022 |
| **total alleles** | **31735** | **271805** | **419735** |
