# Supplementary material for: Machine learning with random subspace ensembles identifies antimicrobial resistance determinants from pan-genomes of three pathogens
Source: PLoS Comput Biol. 2020 Mar 2;16(3):e1007608. doi: 10.1371/journal.pcbi.1007608 (PMC7067475; doi:10.1371/journal.pcbi.1007608)
Supplement: S2 Table — (DOCX) [file pcbi.1007608.s013.docx]

| **S2 Table. AMR phenotypes of PATRIC genomes and corresponding typing methods and standards.** | | | | | | | | | |
| --- | --- | --- | --- | --- | --- | --- | --- | --- | --- |
|  |  | **Phenotype Counts** | | **Typing Method** | | | | **Typing Standard** | |
| **species** | **drug** | **Resistant** | **Susceptible** | **agar dilution** | **disk diffusion** | **MIC** | **Vitek2** | **CLSI** | **EUCAST** |
| *S. aureus* | CIP | 203 | 13 | 0 | 0 | 216 | 0 | 212 | 4 |
| *S. aureus* | CLI | 201 | 20 | 0 | 211 | 10 | 0 | 217 | 4 |
| *S. aureus* | ERY | 201 | 20 | 0 | 211 | 10 | 0 | 217 | 4 |
| *S. aureus* | GEN | 136 | 85 | 0 | 211 | 10 | 0 | 217 | 4 |
| *S. aureus* | SXT | 141 | 80 | 0 | 211 | 10 | 0 | 217 | 4 |
| *S. aureus* | TET | 125 | 96 | 0 | 211 | 10 | 0 | 217 | 4 |
| *P. aeruginosa* | AMK | 114 | 291 | 0 | 0 | 405 | 0 | 405 | 0 |
| *P. aeruginosa* | CAZ | 56 | 18 | 50 | 0 | 24 | 0 | 24 | 50 |
| *P. aeruginosa* | LVX | 213 | 192 | 0 | 0 | 405 | 0 | 405 | 0 |
| *P. aeruginosa* | MEM | 201 | 229 | 25 | 0 | 405 | 0 | 405 | 25 |
| *E. coli* | AMC | 464 | 1058 | 1094 | 1 | 17 | 410 | 17 | 1505 |
| *E. coli* | CAZ | 135 | 1397 | 1094 | 1 | 27 | 410 | 25 | 1507 |
| *E. coli* | CIP | 301 | 1229 | 1094 | 0 | 26 | 410 | 24 | 1506 |
| *E. coli* | GEN | 133 | 1397 | 1094 | 0 | 26 | 410 | 24 | 1506 |
| *E. coli* | IPM | 23 | 1096 | 1094 | 0 | 25 | 0 | 23 | 1096 |
| *E. coli* | TMP | 149 | 263 | 0 | 0 | 2 | 410 | 1 | 411 |
