# Supplementary material for: Machine learning with random subspace ensembles identifies antimicrobial resistance determinants from pan-genomes of three pathogens
Source: PLoS Comput Biol. 2020 Mar 2;16(3):e1007608. doi: 10.1371/journal.pcbi.1007608 (PMC7067475; doi:10.1371/journal.pcbi.1007608)
Supplement: S3 Table — (DOCX) [file pcbi.1007608.s014.docx]

| **S3 Table: Number of significant features associated with antimicrobial resistance in *S. aureus*, as detected by Fisher's Exact tests and Cochran–Mantel–Haenszel tests.** | | | | | | |
| --- | --- | --- | --- | --- | --- | --- |
|  | **Associated with Resistance** | | | **Associated with Susceptibility** | | |
| **Drug** | **Fisher** | **CMH (Bonf.)** | **CMH (B-H)** | **Fisher** | **CMH (Bonf.)** | **CMH (B-H)** |
| CIP | 58 | 20 | 1662 | 608 | 31 | 1263 |
| CLI | 1276 | 0 | 0 | 80 | 0 | 0 |
| ERY | 1436 | 0 | 0 | 90 | 0 | 0 |
| GEN | 2138 | 149 | 1730 | 1892 | 2 | 269 |
| TET | 2117 | 6 | 6 | 1881 | 0 | 0 |
| SXT | 2110 | 22 | 37 | 1876 | 11 | 18 |
| Tests were applied between each antibiotic and each genomic feature (n = number of genomes; n = 216 for CIP, n = 221 for all other drugs). Bonferroni correction was applied to Fisher's Exact tests (FWER ≤ 0.05). Results with either a Bonferroni correction (Bonf, FWER ≤ 0.05) or Benjamini-Hochberg correction (B-H, FDR ≤ 0.05) are shown for the Cochran–Mantel–Haenszel (CMH) tests. Multiple hypothesis corrections were done with m = number of tests = 11485. | | | | | | |
