# Supplementary material for: Machine learning with random subspace ensembles identifies antimicrobial resistance determinants from pan-genomes of three pathogens
Source: PLoS Comput Biol. 2020 Mar 2;16(3):e1007608. doi: 10.1371/journal.pcbi.1007608 (PMC7067475; doi:10.1371/journal.pcbi.1007608)
Supplement: S4 Table — (DOCX) [file pcbi.1007608.s015.docx]

| **S4 Table: Aminoglycoside-modifying enzymes identified by sequence homology in the *P. aeruginosa* pan-genome compared to amikacin resistance phenotypes.** | | | | | |
| --- | --- | --- | --- | --- | --- |
| **Gene** | **Gene Class** | **Count** | **Res.** | **Sus.** | **LOR** |
| AAC(3)-Id | aminoglycoside acetyltransferase | 13 | 11 | 2 | 3.5 |
| AAC(3)-IIc | aminoglycoside acetyltransferase | 2 | 2 | 0 | 3.0 |
| AAC(3)-IIIb | aminoglycoside acetyltransferase | 3 | 0 | 3 | -2.4 |
| AAC(6')-33 | aminoglycoside acetyltransferase | 1 | 1 | 0 | 2.2 |
| AAC(6')-Ib | aminoglycoside acetyltransferase | 1 | 1 | 0 | 2.2 |
| AAC(6')-Ip | aminoglycoside acetyltransferase | 1 | 1 | 0 | 2.2 |
| aadA2 | aminoglycoside adenylyltransferase | 36 | 24 | 12 | 2.6 |
| aadA4 | aminoglycoside adenylyltransferase | 2 | 1 | 1 | 0.9 |
| aadA6/16 | aminoglycoside adenylyltransferase | 5 | 3 | 2 | 1.7 |
| aadA7 | aminoglycoside adenylyltransferase | 6 | 5 | 1 | 3.0 |
| ANT(2'')-Ia | aminoglycoside nucleotidyltransferase | 3 | 1 | 2 | 0.3 |
| APH(3'')-Ib | aminoglycoside phosphotransferase | 5 | 2 | 3 | 0.7 |
| APH(3')-Ia | aminoglycoside phosphotransferase | 1 | 1 | 0 | 2.2 |
| APH(3')-IIb | aminoglycoside phosphotransferase | 400 | 114 | 286 | 3.0 |
| APH(3')-VI | aminoglycoside phosphotransferase | 2 | 2 | 0 | 3.0 |
| APH(6)-Ic | aminoglycoside phosphotransferase | 4 | 2 | 2 | 1.1 |
| APH(6)-Id | aminoglycoside phosphotransferase | 34 | 18 | 16 | 1.6 |
| Log2 odds ratios (LOR) are shown, using weighted pseudocounts to address zeroes in the contingency table (see **Methods** for details). | | | | | |
