# Supplementary material for: Machine learning with random subspace ensembles identifies antimicrobial resistance determinants from pan-genomes of three pathogens
Source: PLoS Comput Biol. 2020 Mar 2;16(3):e1007608. doi: 10.1371/journal.pcbi.1007608 (PMC7067475; doi:10.1371/journal.pcbi.1007608)
Supplement: S5 Table — (DOCX) [file pcbi.1007608.s016.docx]

| **S5 Table. Enrichment for plasmid over chromosomally encoded genetic features selected by SVM-RSE.** For each organism-antibiotic case, the number of plasmid vs. chromosomally encoded features among the top 50 resistance-associated hits was counted, and the odds ratio for plasmid over chromosomal was computed. Rows without a specified drug show the total plasmid and chromosomal feature count for that organism. This analysis was repeated for just non-core genes, where most plasmid features are present. | | | | | | | |
| --- | --- | --- | --- | --- | --- | --- | --- |
|  |  | **Enrichment with respect to   all possible features** | | | **Enrichment with respect to  non-core genes** | | |
| **Organism** | **Drug** | **plasmid** | **chromosomal** | **odds ratio** | **plasmid** | **chromosomal** | **odds ratio** |
| *S. aureus* | - | 144 | 23214 | - | 121 | 2847 | - |
| *S. aureus* | CIP | 0 | 50 | 0 | 0 | 20 | 0 |
| *S. aureus* | CLI | 4 | 46 | 14.39 | 4 | 18 | 5.37 |
| *S. aureus* | ERY | 4 | 46 | 14.39 | 4 | 16 | 6.05 |
| *S. aureus* | GEN | 2 | 48 | 6.80 | 2 | 15 | 3.17 |
| *S. aureus* | SXT | 3 | 47 | 10.49 | 3 | 23 | 3.12 |
| *S. aureus* | TET | 7 | 43 | 27.53 | 7 | 16 | 10.86 |
| *P. aeruginosa* | - | 459 | 178046 | - | 306 | 22849 | - |
| *P. aeruginosa* | AMK | 0 | 50 | 0 | 0 | 27 | 0 |
| *P. aeruginosa* | CAZ | 2 | 48 | 16.36 | 0 | 17 | 0 |
| *P. aeruginosa* | LVX | 0 | 50 | 0 | 0 | 16 | 0 |
| *P. aeruginosa* | MEM | 0 | 50 | 0 | 0 | 27 | 0 |
| *E. coli* | - | 2261 | 187165 | - | 1109 | 36053 | - |
| *E. coli* | AMC | 7 | 43 | 13.51 | 7 | 37 | 6.18 |
| *E. coli* | CAZ | 1 | 49 | 1.69 | 1 | 42 | 0.77 |
| *E. coli* | CIP | 0 | 50 | 0 | 0 | 24 | 0 |
| *E. coli* | GEN | 3 | 47 | 5.29 | 3 | 45 | 2.17 |
| *E. coli* | IPM | 2 | 48 | 3.45 | 2 | 38 | 1.71 |
| *E. coli* | TMP | 1 | 49 | 1.69 | 1 | 39 | 0.83 |
