# Supplementary material for: Machine learning with random subspace ensembles identifies antimicrobial resistance determinants from pan-genomes of three pathogens
Source: PLoS Comput Biol. 2020 Mar 2;16(3):e1007608. doi: 10.1371/journal.pcbi.1007608 (PMC7067475; doi:10.1371/journal.pcbi.1007608)
Supplement: S6 Table — (DOCX) [file pcbi.1007608.s017.docx]

| **S6 Table: Comparison of estimates for *S. aureus, P. aeruginosa,* and *E. coli* core-genome sizes.** | | | | |
| --- | --- | --- | --- | --- |
| **Reference** | **Organism** | **Genomes** | **Core genes** | **Method** |
| This study | *S. aureus* | 288 | 2221 | genes missing in at most 10 genomes |
| Fuchs et al. 2018 [[1]](https://paperpile.com/c/tYJvf1/4WKXB) | *S. aureus* | 32 | 2115 | genes missing in at most 1 genome |
| Bosi et al. 2016 [[2]](https://paperpile.com/c/tYJvf1/6zDyP) | *S. aureus* | 64 | 1441 | Estimated from genome permutations |
| This study | *P. aeruginosa* | 456 | 4700 | genes missing in at most 10 genomes |
| Subedi et al. 2018 [[3]](https://paperpile.com/c/tYJvf1/SB2TD) | *P. aeruginosa* | 22 | 4910 | genes present in >99% of genomes |
| Valot et al. 2015 [[4]](https://paperpile.com/c/tYJvf1/YX48) | *P. aeruginosa* | 17 | 5233 | genes in all genomes |
| Ozer et al. 2014 [[5]](https://paperpile.com/c/tYJvf1/DI1E8) | *P. aeruginosa* | 12 | 5316 | genes in all genomes |
| This study | *E. coli* | 1588 | 3107 | genes missing in at most 10 genomes |
| Kaas et al. 2012 [[6]](https://paperpile.com/c/tYJvf1/KLIlh) | *E. coli* | 186 | 3051 | genes present in >95% of genomes |
| Lukjancenko et al. 2010 [[7]](https://paperpile.com/c/tYJvf1/u5bE2) | *E. coli* | 53 | 1472 | genes in all genomes (excluding Shigella) |
| Rasko et al. 2008 [[8]](https://paperpile.com/c/tYJvf1/UeJ3H) | *E. coli* | 17 | ~2200 | Estimated from genome permutations |
| Previous core genome size estimates are shown, based on varying genome set sizes and definitions of a core gene. Discrepancies between this study and previous studies may be due to either a stricter definition of a core gene (in the case of *S. aureus* or *E. coli*) or a much smaller genome set in the case of *P. aeruginosa*. The method "Estimated from genome permutations" refers to fitting a function (usually exponential) to the size of the core genome vs. number of genomes based on many permutations of genome order. This approach extrapolates the number of core genes present in all genomes for an infinite number of genomes and can be considered the strictest definition of core genome. Detailed references available in S1 Appendix. | | | | |
