# Supplementary material for: Machine learning with random subspace ensembles identifies antimicrobial resistance determinants from pan-genomes of three pathogens
Source: PLoS Comput Biol. 2020 Mar 2;16(3):e1007608. doi: 10.1371/journal.pcbi.1007608 (PMC7067475; doi:10.1371/journal.pcbi.1007608)
Supplement: S7 Table — (DOCX) [file pcbi.1007608.s018.docx]

| **S7 Table: Fisher’s exact test p-values between each COG functional category and the combined core, accessory, or unique genomes of *S. aureus, P. aeruginosa,* and *E. coli*.** | | | |
| --- | --- | --- | --- |
| **COG Functional Category** | **Core** | **Acc** | **Unique** |
| J: Translation, ribosomal structure, biogenesis | <0.00001 | <0.00001 | <0.00001 |
| H: Coenzyme transport/metabolism | <0.00001 | <0.00001 | <0.00001 |
| F: Nucleotide transport/metabolism | <0.00001 | <0.00001 | <0.00001 |
| E: Amino acid transport/metabolism | <0.00001 | <0.00001 | <0.00001 |
| C: Energy production and conversion | <0.00001 | <0.00001 | <0.00001 |
| G: Carbohydrate transport/metabolism | <0.00001 | 0.00047 | <0.00001 |
| I: Lipid transport/metabolism | <0.00001 | <0.00001 | <0.00001 |
| O: PTMs, protein turnover, chaperones | <0.00001 | 0.00013 | <0.00001 |
| P: Inorganic ion transport/metabolism | <0.00001 | 0.31713 | <0.00001 |
| T: Signal transduction mechanisms | <0.00001 | <0.00001 | <0.00001 |
| Q: Secondary metabolites transport/metabolism | <0.00001 | 0.00028 | <0.00001 |
| M: Cell wall/membrane/envelope biogenesis | <0.00001 | 0.33189 | <0.00001 |
| N: Cell motility | <0.00001 | <0.00001 | <0.00001 |
| K: Transcription | <0.00001 | 0.69589 | <0.00001 |
| D: Cell cycle/division, chromosome partitioning | <0.00001 | 0.6214 | <0.00001 |
| U: Intracellular trafficking, secretion, vesicular transport | 0.06502 | 0.00001 | 0.0086 |
| A: RNA processing and modification | 0.66545 | 0.65512 | 0.35414 |
| V: Defense mechanisms | 0.67894 | 0.14832 | 0.10492 |
| L: Replication, recombination and repair | <0.00001 | <0.00001 | 0.13947 |
| S: Function unknown | <0.00001 | <0.00001 | <0.00001 |
| Tests were applied between each COG and gene category (n = number of genes = 72220). COGs are ordered by effect size, as in S9c Figure. | | | |
