# Supplementary material for: Machine learning with random subspace ensembles identifies antimicrobial resistance determinants from pan-genomes of three pathogens
Source: PLoS Comput Biol. 2020 Mar 2;16(3):e1007608. doi: 10.1371/journal.pcbi.1007608 (PMC7067475; doi:10.1371/journal.pcbi.1007608)
Supplement: S8 Table — (DOCX) [file pcbi.1007608.s019.docx]

| **S8 Table: Fisher’s exact test p-values between each COG functional category and the individual core, accessory, and unique genomes of *S. aureus* (SA), *P. aeruginosa* (PA), and *E. coli* (EC).** | | | | | | | | | |
| --- | --- | --- | --- | --- | --- | --- | --- | --- | --- |
|  | **Core Genomes** | | | **Accessory Genomes** | | | **Unique Genomes** | | |
| **COG** | **SA** | **PA** | **EC** | **SA** | **PA** | **EC** | **SA** | **PA** | **EC** |
| **J** | <0.00001 | <0.00001 | <0.00001 | <0.00001 | <0.00001 | <0.00001 | <0.00001 | <0.00001 | <0.00001 |
| **H** | <0.00001 | <0.00001 | <0.00001 | <0.00001 | 0.00002 | 0.00001 | <0.00001 | <0.00001 | <0.00001 |
| **F** | <0.00001 | <0.00001 | <0.00001 | 0.00046 | 0.00001 | 0.00691 | <0.00001 | <0.00001 | <0.00001 |
| **E** | <0.00001 | <0.00001 | <0.00001 | <0.00001 | <0.00001 | 0.0003 | <0.00001 | <0.00001 | <0.00001 |
| **C** | <0.00001 | <0.00001 | <0.00001 | <0.00001 | <0.00001 | 0.19327 | <0.00001 | <0.00001 | <0.00001 |
| **G** | <0.00001 | <0.00001 | <0.00001 | <0.00001 | 0.53968 | <0.00001 | <0.00001 | <0.00001 | <0.00001 |
| **I** | <0.00001 | <0.00001 | <0.00001 | 0.00001 | 0.00094 | 0.96853 | 0.00011 | <0.00001 | <0.00001 |
| **O** | <0.00001 | <0.00001 | <0.00001 | 0.01354 | 0.00489 | 0.5218 | <0.00001 | <0.00001 | <0.00001 |
| **P** | <0.00001 | <0.00001 | <0.00001 | <0.00001 | 0.08958 | 0.4422 | <0.00001 | <0.00001 | <0.00001 |
| **T** | 0.00067 | <0.00001 | <0.00001 | 0.86057 | <0.00001 | 0.00105 | 0.00073 | <0.00001 | <0.00001 |
| **Q** | 0.01269 | <0.00001 | <0.00001 | 0.90916 | 0.0751 | <0.00001 | 0.00933 | <0.00001 | <0.00001 |
| **M** | <0.00001 | <0.00001 | 0.00013 | 0.04235 | 0.05049 | 0.00288 | <0.00001 | <0.00001 | 0.47482 |
| **N** | 1 | <0.00001 | 0.00609 | 0.93916 | 0.9712 | <0.00001 | 0.87672 | <0.00001 | <0.00001 |
| **K** | <0.00001 | <0.00001 | <0.00001 | 0.1288 | 0.29539 | 0.00004 | 0.00116 | <0.00001 | <0.00001 |
| **D** | 0.18291 | 0.00346 | <0.00001 | 0.07709 | 0.78917 | 0.95705 | 0.90148 | 0.01029 | 0.00013 |
| **U** | 0.02353 | 0.45176 | 0.15074 | 0.007 | 0.01099 | <0.00001 | 0.99719 | 0.12774 | 0.00033 |
| **A** | 1 | 0.86136 | 0.44756 | 0.93916 | 0.48628 | 0.91384 | 0.87672 | 0.27756 | 0.83678 |
| **V** | 0.17506 | 0.01038 | 0.1114 | 0.82478 | 0.17517 | 0.93426 | 0.25457 | 0.00164 | 0.45539 |
| **L** | 0.00003 | <0.00001 | <0.00001 | <0.00001 | <0.00001 | <0.00001 | 0.98869 | <0.00001 | 0.00401 |
| **S** | <0.00001 | <0.00001 | <0.00001 | <0.00001 | <0.00001 | 0.00004 | <0.00001 | <0.00001 | <0.00001 |
| Tests were applied between each COG and gene category (n = number of genes; n = 5152 for *S. aureus*, n = 27435 for *P. aeruginosa*, n = 39633 for *E. coli*). COGs are ordered by effect size, as in S9c Figure. | | | | | | | | | |
