# Supplementary material for: Machine learning with random subspace ensembles identifies antimicrobial resistance determinants from pan-genomes of three pathogens
Source: PLoS Comput Biol. 2020 Mar 2;16(3):e1007608. doi: 10.1371/journal.pcbi.1007608 (PMC7067475; doi:10.1371/journal.pcbi.1007608)
Supplement: S1 Appendix — (DOCX) [file pcbi.1007608.s025.docx]

**References for S6 Table**

1. [Fuchs, S. *et al.* AureoWiki ̵ The repository of the Staphylococcus aureus research and annotation community. *Int. J. Med. Microbiol.* **308**, 558–568 (2018).](http://paperpile.com/b/F8GlYw/7XMt)

2. [Bosi, E. *et al.* Comparative genome-scale modelling of Staphylococcus aureus strains identifies strain-specific metabolic capabilities linked to pathogenicity. *Proc. Natl. Acad. Sci. U. S. A.* **113**, E3801–9 (2016).](http://paperpile.com/b/F8GlYw/YEzF)

3. [Subedi, D., Vijay, A. K., Kohli, G. S., Rice, S. A. & Willcox, M. Comparative genomics of clinical strains of Pseudomonas aeruginosa strains isolated from different geographic sites. *Sci. Rep.* **8**, 959 (2018).](http://paperpile.com/b/F8GlYw/KOLAr)

4. [Valot, B. *et al.*](http://paperpile.com/b/F8GlYw/mrGRa) What It Takes to Be a Pseudomonas aeruginosa? The Core Genome of the Opportunistic Pathogen Updated. (2015). doi:10.1371/journal.pone.0126468

5. [Ozer, E. A., Allen, J. P. & Hauser, A. R. Characterization of the core and accessory genomes of Pseudomonas aeruginosa using bioinformatic tools Spine and AGEnt. *BMC Genomics* **15**, 737 (2014).](http://paperpile.com/b/F8GlYw/LdCEe)

6. [Kaas, R. S., Friis, C., Ussery, D. W. & Aarestrup, F. M. Estimating variation within the genes and inferring the phylogeny of 186 sequenced diverse Escherichia coli genomes. *BMC Genomics* **13**, 577 (2012).](http://paperpile.com/b/F8GlYw/Lg0Vq)

7. [Lukjancenko, O., Wassenaar, T. M. & Ussery, D. W. Comparison of 61 sequenced Escherichia coli genomes. *Microb. Ecol.* **60**, 708–720 (2010).](http://paperpile.com/b/F8GlYw/XZo8a)

8. [Rasko, D. A. *et al.* The pangenome structure of Escherichia coli: comparative genomic analysis of E. coli commensal and pathogenic isolates. *J. Bacteriol.* **190**, 6881–6893 (2008).](http://paperpile.com/b/F8GlYw/TEnp7)
