# Supplementary material for: Machine learning with random subspace ensembles identifies antimicrobial resistance determinants from pan-genomes of three pathogens
Source: PLoS Comput Biol. 2020 Mar 2;16(3):e1007608. doi: 10.1371/journal.pcbi.1007608 (PMC7067475; doi:10.1371/journal.pcbi.1007608)
Supplement: S1 Text — (DOCX) [file pcbi.1007608.s026.docx]

**Machine learning with random subspace ensembles identifies antimicrobial resistance determinants from pan-genomes of three pathogens**

Jason C. Hyun, Erol S. Kavvas, Jonathan M. Monk and Bernhard O. Palsson

**Supplemental Discussion**

*Analysis of genetic diversity in S. aureus, P. aeruginosa, and E. coli pan-genomes*

For each pan-genome, genes were classified based on how frequently they were observed: core (missing in 0-10 genomes), accessory (missing in >10 genomes, present in >10 genomes), or unique (present in 1-10 genomes), (S1 Table, S9a Fig). This classification found 2221, 4700, and 3107 genes to be core genes for *S. aureus*, *P. aeruginosa*, and *E. coli*, respectively, which is consistent with previously observed core-genome sizes when accounting for the number of genomes examined and different thresholds for identifying core genes (S6 Table).

To quantify pan-genome openness, or the propensity for new genomes to carry previously unidentified genes, the relationship between the number of genomes and the new gene rate was modeled using Heap’s Law [[1]](https://paperpile.com/c/7Tf2gl/mEbNM). From 2000 random permutations of genome order for each pan-genome, the Heap’s Law exponent, α (in which a value of 1 represents a closed pan-genome and 0 represents a completely open pan-genome), was estimated as 0.83 for *S. aureus* and 0.71 for both *P. aeruginosa* and *E. coli* (S9b Fig); this result is consistent with previous observations that *S. aureus* harbors a relatively closed pan-genome [[1]](https://paperpile.com/c/7Tf2gl/mEbNM).

To examine differences in function between core, accessory, and unique genes, eggNOG-mapper [[2]](https://paperpile.com/c/7Tf2gl/57cK) was used to assign a Clusters of Orthologous Groups (COG) functional category to every gene in each pan-genome. We find that the distributions of gene functions in the three core genomes are very similar, as well as for the three accessory genomes and three unique genomes (S10 Fig, S11 Fig); in other words, a gene’s function is associated with its frequency across multiple organisms. This observation was quantified by computing log2 odds ratios (LORs) between the core/accessory/unique gene sets and each COG functional category (S9c Fig).

We find several functions to be enriched in either the core or non-core genomes for all three organisms (S7 Table, S8 Table). Genes related to translation, ribosomal structure, energy production/conversion, or the transport/metabolism of core metabolites were more likely to be found in the core genome (p<0.00001), while genes related to DNA replication/recombination/repair or have unknown function were more likely to be found among accessory genes (p<0.00001). The core-enriched functions recapitulate the essentiality of translation and core metabolism for survival, while non-core-enriched functions appear to be more niche-specific (non-core genes involved in DNA manipulation were comprised primarily of mobile elements). Ultimately, it appears that different organisms allocate similar fractions of their genes towards specific functional groups, at least among genes that have been functionally characterized.

**Supplemental Discussion - Methods**

*Comparison of pan-genome size and openness*

Genes were categorized by frequency: for a pan-genome of n genomes, a gene is 1) core, if found in all n genomes, 2) near-core, if missing from at most 10 and at least 1 genomes, 3) accessory, if found in at least 11 genomes and missing from at least 10 genomes, 4) near-unique, if found in at least 2 genomes and at most 10 genomes, or 5) unique, if found in exactly 1 genome (S9a Fig). Subsequent analyses combine core/near-core as just “core”, and unique/near-unique as just “unique”. Pan-genome openness was estimated using Heap’s law,


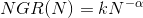


where N is the number of genomes, NGR is the new gene rate, or number of new genes introduced per genome, and k and α are fitted parameters [[1]](https://paperpile.com/c/7Tf2gl/mEbNM). NGR was estimated as the median NGR from permuting the order of genomes 2000 times, and Heap’s law parameters were fitted by linear regression between log(NGR) and log(N) (S9b Fig).

*Functional characterization of pan-genomes*

All alleles from all three pan-genomes were assigned a Clusters of Orthologous Groups (COG) functional category using the public eggNOG-mapper v1 server [[2]](https://paperpile.com/c/7Tf2gl/57cK). Each gene cluster was assigned the majority COG category of its alleles, weighted by the number of genomes containing each allele. Rare instances in which genes had no majority COG or had a mixture of multiple COGs assigned were ignored, and COG distributions were visualized with and without the “S: Function Unknown” category (S10 Fig).

To assess the stability of functional distributions, genes within each pan-genome were categorized as core, accessory, or unique based on a similar threshold X from the pan-genome size analysis: genes missing in up to X genomes were labeled core, genes in up to X genomes were labeled unique, and all other genes were labeled accessory. The distribution of COG categories for core, accessory, and unique genes were plotted as X increased from 1 to 10 (S11 Fig). For enrichment analysis, the log2 odds ratio (LOR) and Fisher’s exact test p-value between each COG category and each gene subset (core, accessory, unique) were computed with the threshold X = 10 (S9c Fig, S7 Table, S8 Table). LORs for a given COG category and gene subset were computed as follows:


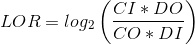


where CI is the number of genes with the COG in the gene subset, CO is the number of genes with the COG outside the gene subset, DO is the number of genes with a different COG outside the gene subset, and DI is the number of genes with a different COG in the gene subset. Undefined LORs were replaced with 0, as all such cases had very few genes with which to examine significant enrichment. The category “B: Chromatin structure and dynamics” was ignored for this analysis as only one gene was annotated with that functional category.

**Supplemental Discussion - References**

1. [Tettelin H, Riley D, Cattuto C, Medini D. Comparative genomics: the bacterial pan-genome. Curr Opin Microbiol. 2008;11: 472–477.](http://paperpile.com/b/7Tf2gl/mEbNM)

2. [Huerta-Cepas J, Forslund K, Coelho LP, Szklarczyk D, Jensen LJ, von Mering C, et al. Fast Genome-Wide Functional Annotation through Orthology Assignment by eggNOG-Mapper. Mol Biol Evol. 2017;34: 2115–2122.](http://paperpile.com/b/7Tf2gl/57cK)
