# Supplementary material for: Machine learning with random subspace ensembles identifies antimicrobial resistance determinants from pan-genomes of three pathogens
Source: PLoS Comput Biol. 2020 Mar 2;16(3):e1007608. doi: 10.1371/journal.pcbi.1007608 (PMC7067475; doi:10.1371/journal.pcbi.1007608)
Supplement: Attachment — Submitted filename: Response_to_Reviewers.docx [file pcbi.1007608.s027.docx]

**Response to Reviewers**

***Reviewer #1***: In this submitted manuscript the authors (Hyun et al.) propose a method that predicts antimicrobial resistant (AMR) genes using SVM ensembles trained using random subspace ensemble (RSE). The SVM-RSE predictors were built for 3 of the main multi-drug resistant Bacterial species and 10 antibiotic classes extracting features from the 3 pan-genomes.

The authors show that the SVM-RSEs are more accurate in predicting AMR genes than well-established association tests. Furthermore the technique is reference-agnostic and has the potential to be improved further.

The manuscript is generally nice to read and has ample supporting material.

We thank the reviewer for carefully evaluating our manuscript and for their compliments on the manuscript’s readability. We have corrected all of the minor issues the reviewer identified in this resubmission.

Following is a list of minor issues that I encountered reading the manuscript:

[1.1] I) In lines 229-231 the authors state that oversampling or undersampling did not affect the performance. this does not seem so clear from Fig3b, were ranks for SVM-RSE-O and SVM-RSE-U are sensibly higher than those given from SVM-RSE. Please rephrase accordingly or explain in more details.

Thank you for this comment. From the rankings, we find that there are a mixture of known AMR genes detected at both higher and lower rankings by SVM-RSE-O/U compared to SVM-RSE, resulting in a small overall effect is small. We have updated our discussion of SVM-RSE-O and SVM-RSE-U to include this (line 200)

[1.2] II) In figures (main and supplementary) change "# of X" with "Number of X"

Corrections made in Fig 4, Fig S1, Fig S2 (previously Fig S5), Fig S9 (previously Fig 1), and Fig S11 (previously Fig S3).

[1.3] III) In Fig. 1 it would be nice to have a more descriptive legend (e.g. near-core: missing from <= 10 strains) Legend for Fig S9a (previously Fig 1a) now includes strain counts for each gene category.

[1.4] IV) In Fig2a, specify the distance used in the y-axis

Relabeled y-axis “distance” to “Jaccard distance of genetic features” for Fig 1a (previously Fig 2a)

[1.5] V) In Fig2b and c, add a legend for the resistance patterns

Added legend for Fig 1b-c (previously Fig 2b-c), changed color of subtype plot to distinguish from AMR phenotype plot.

[1.6] VI) Fig. S2, and S3: add "Functional category" above the legends

Added legend labels to Fig S10 and Fig S11 (previously Fig S2 and Fig S3)

[1.7] VII) Lines 173-175: there is probably a missing/wrong word in the sentence (e.g. "or are involved efflux span", please fix it. Fixed

[1.8] VIII) Fig 3b: in the x-axis change "ImsR" with "ImrS" Fixed

***Reviewer #2:*** In this work, Hyun et al. have developed a Support Vector Machine (SVM)-based method to identify and predict antimicrobial resistance determinants from (pan)genomic data.

First, they build pangenomes for three species of interest (S. aureus, P. aeruginosa and E. coli) and then they use such gene sets to train and apply models for AMR determinants search.

They succeed in the identification of known determinants and their method seems to outperform common association tests and to achieve prediction accuracies competitive with

previous machine learning approaches (although a proper and clear benchmarking is missing in the current submission). The paper is well written and the amount of data analyses is notable.

Overall I have a few concerns regarding the structure of the paper and the lack of experimental validation on some of the predictions.

- [2.1] The first part of the results is devoted to “standard” pangenomic analyses. It is no news that core and accessory gene sets do not show interesting and significantly enriched COG categories. Those categories are to broad, especially for the purpose of the current analysis. I would suggest to move the first paragraph (“Analysis of genetic diversity in S. aureus, P. aeruginosa, and E. coli pan-genomes”) to SM and start the Results section from the current “Support vector machine ensembles identify known AMR genes more…”.This would allow going straight to the point.

We thank the reviewer for this valuable suggestion and agree that the section on pan-genome analyses doesn’t fit with the rest of the manuscript. In this resubmission we have moved the bulk of that section to the supplementary material. The manuscript now starts with a brief description of using pan-genomes for feature engineering necessary for our SVM approach.

- [2.2] It is interesting that the SVM-RSE method identifies novel putative AMR determinants. This, however, calls for an experimental validation of the capability of a few of such genes (ideally one for each of the species) to confer resistance to the organism harboring them. This would render the manuscript and the implemented approach much more reliable. Do those substitutions confer resistance to otherwise susceptible strains? Additionally, hflX or oppD are ideal targets to validate the predictions made by the authors.

We have searched the literature for validations of these novel putative AMR determinants and found additional information supporting our predictions for four of our strongest candidates (*hflX-*erythromycin [[1]](https://paperpile.com/c/1qmwpL/97orc), *oppD*-gentamicin [[2]](https://paperpile.com/c/1qmwpL/tV1zA), *ahpF-gentamicin* [[3](https://paperpile.com/c/1qmwpL/nYoPP),[4]](https://paperpile.com/c/1qmwpL/6wXTo), and *repL*-clindamycin [[5]](https://paperpile.com/c/1qmwpL/TdqmM)). Furthermore, we feel that the correct identification of 45 known AMR determinants further validates our approach. While we agree that experimental validations would be valuable unfortunately they are out of the scope of the current manuscript. We hope that other, experimentally focused research labs will take our list of predictions for future validations. As suggested by reviewer 3 we have now listed these predictions in an easily accessible format to facilitate future validation experiments (Dataset S3 and Dataset S4 for sequences and annotations, respectively).

- [2.3] I have a (minor) concern about the criterion used to classify a gene into core, near-core, accessory, unique or near-unique. The authors have shown a threshold based on a fixed number of strains (10, page 18, from line 410). However, the genome datasets are highly variable among the selected species (288, 456, and 1588 publicly-available genomes for S. aureus, P. aeruginosa, and 96 E. coli, respectively) so I think It would be worth replacing such a fixed threshold with a relative amount (10%?). I would be curious to see whether the numbers and the results would change applying such a relative criterion.

We agree that the criterion used for distinguishing core from non-core genes is something that could be developed further in future works. Our rationale for using a fixed threshold was based on plotting the number of genes missing from at most 0 strains, at most 1 strain, at most 2 strains, etc. for each pan-genome and identifying the inflection point, which was coincidently about 10 strains for all three species (Fig S1). In order to evaluate the validity of this approach and its effect on downstream calculations we performed a sensitivity analysis by re-running our SVM-RSE approach using two relative thresholds as the reviewer suggested (2% and 10%, line 270). This analysis allows us to examine the robustness of selected features to the selected threshold. We find that the top 100 features (top 50 resistance-associated + top 50 susceptible-associated) are reasonably consistent between the three thresholds across all 16 organism x drug cases, with an average Jaccard similarity of 0.744 when comparing thresholds of 10 vs 10%, and 0.818 when comparing thresholds of 10 vs 2% (new Fig S5). Interestingly, the least consistent cases did not necessarily coincide well with either cases where the thresholds differed the most such as *E. coli* 10 vs 10% nor cases where the model performed the worst (new Fig 3). One factor that may be contributing to this discrepancy may be the relatively complexity of resistance mechanisms to a particular drug. For instance, the three fluoroquinolone cases had lower consistency between thresholds; as resistance is reasonably well explained by mutations in just two genes (*gyrA* and *parC*), a larger fraction of other top hits may be noise or features with weaker signal, and thus less consistently detected.

**Figure S5. Consistency of selected features for different core gene thresholds.** The top 100 features (top 50 resistance-associated + top 50 susceptibility-associated) were identified using SVM-RSE for three different core gene thresholds (10: missing from at most 10 genomes, 10%: missing from at most 10% of all genomes, 2%: missing from at most 2% of all genomes). For each pair of thresholds, the fraction of shared vs. non-shared features in the union of their top 100 feature sets were computed. Non-shared features were classified as either “not shared”, where both representations contain the feature, or “diff. coded”, where the feature is only available under one of the thresholds.

We have also made available figure-associated data in tabular format in Dataset S5 (specifically, for Fig 1b, Fig 1c, Fig 4, Fig S2b-c, Fig S5, Fig S6a-b, and Fig S9c).

***Reviewer #3***: Hyun and colleagues provide a novel machine learning-based approach for finding enriched sets of alleles involved in AMR. These are compared to known AMR gene alleles, and some discussion is provided for the genes that are implicated by the models that were previously unrecognized in these species. The paper is well written and I enjoyed reading it. However, there were several places in the manuscript where I became very confused. In my opinion text requires a major revision that centers on clarifying certain points, and directly focusing on the message of the paper. That being said, I think that it is scientifically sound and that this paper will be of importance to readers.

We are glad that the reviewer enjoyed reading the manuscript and appreciate the comments related to clarifying certain sections of the manuscript. We feel that that newly structured manuscript is less confusing, more readable and ultimately has benefited greatly from the reviewer’s comments.

Here are my comments:

[3.1] In the intro, has this RSE approach been used with success elsewhere? Was the choice inspired in some way or invented by the authors?

Thank you for this comment. Yes, the first found biological application of RSE is in imaging [[6]](https://paperpile.com/c/1qmwpL/Z5Gc). In the original submission we referenced this on line 88, and in this re-submission we have added additional details within the intro.

[3.2] The last paragraph of the intro, line 88, says that RSEs aggregate classifiers trained on random subsets of both the sample set and the feature set. This requires a sentence explaining what the sample set and feature set are in this context. At first I thought samples were genomes, but since this is a study of pan genomes that was not clear (at this point in the paper).

The samples are indeed genomes. The matrix used to train all classifiers is of shape m x n with m=number of samples (genomes) and n = number of features (features being presence of absence of a given genetic feature, ie. gene or specific allele). We have added text to clarify this point in the main manuscript (line 87).

[3.3] Figure 1a says strains, but I think the authors mean genomes since two genomes of the same strain could exist in the training set.

We thank the reviewer for this point, its true that we are building our pan-genome out of genomes and that it's possible that one strain could potentially have two or more genome sequences associated with it. We have edited the caption for Fig 1a, labels for Fig 1c (now Fig S9), and labels in Fig 2a (previously Fig 3a) to correct this point. We have also updated the text and captions/labels for other supplementary material to refer to our samples as genomes rather than strains.

[3.4] Did the authors do anything to account for the phylogenetic distribution of the strains of each species?

We evaluated the effect of phylogenetic distribution in our initial benchmarking with the *S. aureus* dataset by examining whether the SVM-RSE algorithm could more reliably detect AMR genes if we balanced the representation of each strain cluster we found in Fig 2, by either oversampling smaller clusters or undersampling larger clusters (called SVM-RSE-O and SVM-RSE-U, respectively). We found that these adjustments neither improved nor reduced the performance of SVM-RSE in a consistent manner (Fig 3b), (line 200) and ultimately we did not adjust for phylogenetic distribution in our final results. Based on the outcome of this analysis we are confident that SVM-RSE in its simplest form is already reasonably robust to phylogenetic distribution.

[3.5] Step 4 of figure 3a says “Train SVMs on AMR phenotypes, average weights”. I can’t find exactly how the authors processed the AMR phenotypes. In the case of MICs, are they predicting MICs? Was everything converted to susceptible vs. resistant, S vs. I vs. R, susceptible vs. non-susceptible etc. If so, did they use CLSI or EUCAST?

Thank you for pointing out these missing details. We used PATRIC’s determination of susceptible and resistant phenotypes to train our models, and have added Table S2 that summarizes the typing methods and standards involved in our dataset. For all organism-antibiotic cases examined, we converted everything to binary AMR phenotypes (Susceptible vs. Intermediate/Resistant, which are the three most common annotations available on PATRIC), and have updated the methods to make this more clear (line 469). We used this approach as MIC values were only available for a small fraction of the dataset, and the “Intermediate” phenotype was also too rare (0%-7.2% per case, average 1.8% across all cases) to treat as a separate category. Finally, with the exception of the *P. aeruginosa-*ceftazidime case, all other cases were either >94% CLSI or >97% EUCAST.

| **Table S2. AMR phenotypes of PATRIC genomes and corresponding typing methods and standards.** | | | | | | | | | |
| --- | --- | --- | --- | --- | --- | --- | --- | --- | --- |
|  |  | **Phenotype Counts** | | **Typing Method** | | | | **Typing Standard** | |
| **species** | **drug** | **Resistant** | **Susceptible** | **agar dilution** | **disk diffusion** | **MIC** | **Vitek2** | **CLSI** | **EUCAST** |
| *S. aureus* | CIP | 203 | 13 | 0 | 0 | 216 | 0 | 212 | 4 |
| *S. aureus* | CLI | 201 | 20 | 0 | 211 | 10 | 0 | 217 | 4 |
| *S. aureus* | ERY | 201 | 20 | 0 | 211 | 10 | 0 | 217 | 4 |
| *S. aureus* | GEN | 136 | 85 | 0 | 211 | 10 | 0 | 217 | 4 |
| *S. aureus* | SXT | 141 | 80 | 0 | 211 | 10 | 0 | 217 | 4 |
| *S. aureus* | TET | 125 | 96 | 0 | 211 | 10 | 0 | 217 | 4 |
| *P. aeruginosa* | AMK | 114 | 291 | 0 | 0 | 405 | 0 | 405 | 0 |
| *P. aeruginosa* | CAZ | 56 | 18 | 50 | 0 | 24 | 0 | 24 | 50 |
| *P. aeruginosa* | LVX | 213 | 192 | 0 | 0 | 405 | 0 | 405 | 0 |
| *P. aeruginosa* | MEM | 201 | 229 | 25 | 0 | 405 | 0 | 405 | 25 |
| *E. coli* | AMC | 464 | 1058 | 1094 | 1 | 17 | 410 | 17 | 1505 |
| *E. coli* | CAZ | 135 | 1397 | 1094 | 1 | 27 | 410 | 25 | 1507 |
| *E. coli* | CIP | 301 | 1229 | 1094 | 0 | 26 | 410 | 24 | 1506 |
| *E. coli* | GEN | 133 | 1397 | 1094 | 0 | 26 | 410 | 24 | 1506 |
| *E. coli* | IPM | 23 | 1096 | 1094 | 0 | 25 | 0 | 23 | 1096 |
| *E. coli* | TMP | 149 | 263 | 0 | 0 | 2 | 410 | 1 | 411 |

[3.6] Similarly, this looks like they built an ensemble of SVMs for predicting the key features for one antibiotic at a time, analyzing one species at a time, but that also is not clear until you get very deep into the paper and it is not made clear in the methods.

We did evaluate one species at a time as we were most interested in the method as opposed to identifying AMR related genes that were consistent across all three species. Given that the species examined include both Gram positives and Gram negatives we do not expect them to possess similar mechanisms. We’ve updated our description of the workflow in both the results (line 143) and the methods (line 516, 542) and updated Fig 2a (previously Fig 3a) to make it clear that we are comparing the species and antibiotics individually.

[3.7] For any core gene, each mutational variant observed would create another dimension in the vector. Some important core genes would be expected to have very few variants and hence dimensions, and some might have many. Do the authors have any insights on how this might affect accuracy or the ultimate selection of AMR-related features in either a regular SVM or a RSE? Are the important genes, simply those that have few variants?

Thank you for the suggestion to investigate this bias in feature selection. Initially, we agreed that the meaningful core gene variants selected by machine learning should be of core genes with relatively few variants, since the alternative is having many relatively rare variants that individually have weak statistical signal. However, we instead found that there is a slight bias for core genes with more variants to be selected, though this enrichment is very modest in even the most strongly biased cases for each organism (new Fig S6, line 283). The level of bias is also fairly consistent across different drug cases for a given organism, regardless of the accuracy of the corresponding model. Ultimately, it does not appear that the number of variants observed for a given core gene significantly impacts the chance that it is selected by machine learning. One possible explanation for this bias towards core genes with more variants may be that mutations in core genes are more often necessary for increases in resistance and thus there may be more variants established in a population exploring these possibilities. Resistance increases fitness (however small) against exposure to a given antimicrobial, resulting in more variants established in the population.

**Figure S6. Overall sequence variability of selected core gene alleles.** For each organism-antibiotic case, the distribution of the number of alleles of all core genes was compared to that of core genes for which at least one allele was selected by SVM-RSE to be associated with resistance or susceptibility. The (a) mean and (b) median of the selected core gene allele count is shown for each case, compared to the mean for all core genes of the corresponding species (dotted lines). For each species, the allele count distributions are shown for the case with the largest difference in mean allele count, (d) *S. aureus* vs. sulfamethoxazole/trimethoprim, (e) *P. aeruginosa* vs. amikacin, and (f) *E. coli* vs. ceftazidime.

[3.8] Coloring and/or scale should be defined in Figure 1c.

Colorbar of Fig S9c (previously Fig 1c) has been labeled “LOR”.

[3.9] The authors use the term allele, throughout, which I believe means alternative forms of the same gene. However, on line 166 they jump to protein space by defining alleles based on blastp. Is the feature vector based on proteins or genes? I think it is proteins. They should clarify, and I recommend careful use of terms here. This also makes Table 3 a bit confusing because I think the table is describing nucleotide SNPs in column 2 (ie. gene space), but I don’t understand how the model is identifying a nucleotide here if it is based on proteins.

Thank you for pointing out this issue, we have added text early in our analysis description (line 111) that defines our use of “allele” as a unique amino acid sequence variant. Unique amino acid sequence variants are manifested as unique genes or ‘alleles’ on the genome as well, hence our use of this term. The reviewer is correct, however, that moving to nucleic acid space would enable analysis of even more potential alleles. However, in this manuscript we sought to stick to unique protein variants to limit the number of features and to focus on alleles that would manifest as distinct changes in the final protein. All the analysis in this work is in protein space, and column 2 in Table 3 refers to arbitrarily assigned names for unique amino acid sequences for *gyrA* and *parC* observed in the pan-genome, with their respective substitutions/deletions described in column 5.

[3.10] Along these lines, the results section starting on line 181 sets up a comparison of statistical tests vs. the SVM method. Some lead in is required here. I think that the authors are performing statistics over the same matrix that they are performing the svm analysis but that isn’t clear in the text.

Thank you for this suggestion, we have added text more clearly introducing the rationale and setup for comparing machine learning models and statistical tests as GWAS methods (line 141).

[3.11] Based on my recollection, I think that set of antimicrobials and their associated alleles for the 3 species tends to skew toward chromosomally encoded AMR genes (Lines 162-165). Is that the case? I don’t think that action is required in terms of the experimental approach but that should be mentioned, with some discussion of implications, if it is indeed true.

We thank the reviewer for this valuable perspective on interpreting our selected features. We have identified the genetic features for each species as plasmid or chromosomal by mapping contigs to known plasmids on PLSDB (see Methods for details), and computed the enrichment for plasmid-encoded features among the top 50 resistance-associated features for each organism-antibiotic case (new Table S5, line 292). In a majority of cases, the selected features were actually enriched for plasmid-features, with two major exceptions: 1) All three fluoroquinolone cases did not select any plasmid features, likely because the main mechanism of resistance is mutation of the targeted chromosomal genes *gyrA* and *parC*. 2) *P. aeruginosa* cases did not select any plasmid features; we suspect this is due to a notably smaller number of features that could be identified as plasmid for this species (1.3% of non-core genes, compared to 4.1% for *S. aureus* and 3.0% for *E. coli*).

| **Table S5. Enrichment for plasmid over chromosomally encoded genetic features selected by SVM-RSE.** For each organism-antibiotic case, the number of plasmid vs. chromosomally encoded features among the top 50 resistance-associated hits was counted, and the odds ratio for plasmid over chromosomal was computed. Rows without a specified drug show the total plasmid and chromosomal feature count for that organism. This analysis was repeated for just non-core genes, where most plasmid features are present. | | | | | | | |
| --- | --- | --- | --- | --- | --- | --- | --- |
|  |  | **Enrichment with respect to   all possible features** | | | **Enrichment with respect to  non-core genes** | | |
| **Organism** | **Drug** | **plasmid** | **chromosomal** | **odds ratio** | **plasmid** | **chromosomal** | **odds ratio** |
| *S. aureus* | - | 144 | 23214 | - | 121 | 2847 | - |
| *S. aureus* | CIP | 0 | 50 | 0 | 0 | 20 | 0 |
| *S. aureus* | CLI | 4 | 46 | 14.39 | 4 | 18 | 5.37 |
| *S. aureus* | ERY | 4 | 46 | 14.391 | 4 | 16 | 6.05 |
| *S. aureus* | GEN | 2 | 48 | 6.80 | 2 | 15 | 3.17 |
| *S. aureus* | SXT | 3 | 47 | 10.49 | 3 | 23 | 3.12 |
| *S. aureus* | TET | 7 | 43 | 27.53 | 7 | 16 | 10.86 |
| *P. aeruginosa* | - | 459 | 178046 | - | 306 | 22849 | - |
| *P. aeruginosa* | AMK | 0 | 50 | 0 | 0 | 27 | 0 |
| *P. aeruginosa* | CAZ | 2 | 48 | 16.36 | 0 | 17 | 0 |
| *P. aeruginosa* | LVX | 0 | 50 | 0 | 0 | 16 | 0 |
| *P. aeruginosa* | MEM | 0 | 50 | 0 | 0 | 27 | 0 |
| *E. coli* | - | 2261 | 187165 | - | 1109 | 36053 | - |
| *E. coli* | AMC | 7 | 43 | 13.51 | 7 | 37 | 6.18 |
| *E. coli* | CAZ | 1 | 49 | 1.69 | 1 | 42 | 0.77 |
| *E. coli* | CIP | 0 | 50 | 0 | 0 | 24 | 0 |
| *E. coli* | GEN | 3 | 47 | 5.29 | 3 | 45 | 2.17 |
| *E. coli* | IPM | 2 | 48 | 3.45 | 2 | 38 | 1.71 |
| *E. coli* | TMP | 1 | 49 | 1.69 | 1 | 39 | 0.83 |

[3.12] In figure 2A and B I don’t understand how the data can cluster if there is no information on susceptible vs. resistant phenotypes (2B) for many of the strains in the purple clade on the right-hand side of the plot. Were the purple strains used in the model?

Thank you for pointing out this ambiguity, we have updated the text to make this more clear (line 146). In Fig 1a (previously Fig 2a), strains are clustered solely on the basis of gene and allele content (the matrix in Fig 2a, previously Fig 3a). The resulting clustering happens to group strains together by similar AMR phenotype and subtype metadata available from PATRIC. The purple strains were included in our dataset for the construction and analysis of the *S. aureus* pan-genome, but aside from the few with appropriate AMR phenotype data, were indeed not involved in the training or evaluation of any AMR models.

[3.13] The rationale for the computation of pan-genome openness (line 120) and its depiction in Figure 1 and the supplemental figure are not entire clear to me. If it is required, please clarify. If not, it might help the paper if this went in the supplement. This is also true of the extensive examination of overlap in function between core and accessory genes. This is important but not of primary importance, unless I have missed something.

We thank the reviewer for this valuable suggestion and agree that the section on pan-genome analyses doesn’t fit with the rest of the manuscript. In this resubmission we have moved the bulk of this section to the supplementary material. The manuscript now starts with a brief description of using pan-genomes for feature engineering necessary for our SVM approach.

[3.14] The authors previously wrote a really nice paper on M. tuberculosis (ref 6). Why was this organism excluded from the analysis? Is there something different about this organism that makes it less amenable to the method?

We thank the reviewer for their appreciation of our previous paper on *M. tuberculosis*. We sought to expand that analysis to the study of Gram negative pathogens as well as to pathogen species with much more open pan-genomes. More open pan-genomes meant that there was a much larger feature space compared to *M. tuberculosis* and thus we needed to adopt new approaches such as RSE in order to robustly analyze the data. We don’t expect *M. Tuberculosis* to be less amenable to the approach presented here but included *S. aureus* as a representative gram-positive organisms with a more closed pan-genome .

[3.15] I understand that shifting from an optimization for model accuracy to a focus on gene identification is important, but the accuracy of the models should still be described in greater detail. It only appears on line 265. I think that the precision vs. recall data are still important to this study because they will provide information that the models are correctly balanced and capable of predicting both classes accurately. This in turn lends support to the idea that we should believe the feature list that has been implicated in AMR. To this end, I think that Figure S4 (counts of SR genomes) and S7 (Accuracy vs MCC) should be brought back into the main text, and that either precision and recall, or F1 should be showed as well.

We appreciate the reviewer’s suggestions to expand on the accuracy of the SVM-RSE approach. We have computed the average Accuracy/MCC/Precision/Recall/AUROC from 5-fold CV for each of the 16 species x drug cases, reported those results along with counts as a single main figure (new Fig 3, line 256), and removed the redundant figures (previously Fig S4 and S7). We have also plotted ROC curves for each case (new Fig S4). We confirmed that the cases that perform below average by any of the newly calculated metrics were already highlighted by the low MCCs computed for our original manuscript.

**Figure 3. Predictive performance of SVM-RSE on 16 organism-antibiotic cases.** (a) Distribution of AMR phenotypes for each case. Organisms examined are S. aureus (SA), P. aeruginosa (PA), and E. coli (EC). Antibiotics examined are ciprofloxacin (CIP), clindamycin (CLI), erythromycin (ERY), gentamicin (GEN), tetracycline (TET), sulfamethoxazole/trimethoprim (SXT), amikacin (AMK), ceftazidime (CAZ), levofloxacin (LVX), meropenem (MEM), amoxicillin/clavulanic acid (AMC), imipenem (IPM), and trimethoprim (TMP). (b) SVM-RSE performance metrics from 5-fold cross validation. Performance values shown are averages and standard errors from 5-fold cross validation. The left-most column “log2(R/S)” shows the extent of class imbalance, the log2 of the number of resistant genomes / number of susceptible genomes.

[3.16] It seems like this method casts a wide net. That is, the top features are enriched by known AMR variants, but that there is still a lot of genes that are being pulled in and that some analysis is required to determine their biological relevance. It would be nice to get these lists. It would bring interest to the bench scientists.

Thank you for this valuable suggestion. In our resubmission we have added new datasets containing the protein sequences (Dataset S3) and annotations (Dataset S4) for the top 50 resistance-associated features for each organism-antibiotic case. We agree that this list will be very useful to bench scientists and hope that readers will pursue experimental studies to confirm several of the predictions made here.

Small things:

[3.17] Line 154 define DHFR

Expanded to “dihydrofolate reductase”

[3.18] Line 249, I think the authors mean trimethoprim/sulfamethoxazole, but I’m not 100% sure.
For *E. coli*, we examined resistance trimethoprim alone because data for the combined therapy was sparse. For *S. aureus*, we did indeed examine resistance to combined the trimethoprim/sulfamethoxazole.

[3.19] Line 265 “an accuracies” - Fixed

[3.20] In Dataset S1, the underscores in the PATRIC ids should be converted to periods, or a table legend is required. Might be good to convert cells to text format first. - Fixed

**Updated Labels for Revised Figures, Tables, and other data (in order of appearance)**

| **Updated Label** | **Old Label** |
| --- | --- |
| S1 Dataset | S1 Dataset |
| S1 Fig | S1 Fig |
| S1 Table | [New] |
| S2 Table | [New] |
| S2 Dataset | S2 Dataset |
| Table 1 | Table 1 |
| S2 Fig | Fig S5 |
| Fig 1 | Fig 2 |
| Fig 2 | Fig 3 |
| S3 Table | Table S4 |
| Fig 3 | [New] |
| Table 2 | Table 2 |
| S4 Table | Table S5 |
| S3 Fig | Fig S6 |
| S4 Fig | [New] |
| S5 Fig | [New] |
| S6 Fig | [New] |
| S5 Table | [New] |
| Table 3 | Table 3 |
| S7 Fig | S8 Fig |
| S8 Fig | S9 Fig |
| S3 Dataset | [New] |
| S4 Dataset | [New] |
| Table 4 | Table 4 |
| Fig 4 | Fig 4 |
| S9 Fig | Fig 1 |
| S6 Table | S1 Table |
| S10 Fig | S2 Fig |
| S11 Fig | S3 Fig |
| S7 Table | S2 Table |
| S8 Table | S3 Table |
| S5 Dataset | [New] |
| S1 Appendix | S1 Appendix |

**Response to Reviewers - References**

1.	[Duval M, Dar D, Carvalho F, Rocha EPC, Sorek R, Cossart P. HflXr, a homolog of a ribosome-splitting factor, mediates antibiotic resistance. Proc Natl Acad Sci U S A. 2018;115: 13359–13364.](http://paperpile.com/b/1qmwpL/97orc)

2.	[Dorer MS, Fero J, Salama NR. DNA damage triggers genetic exchange in Helicobacter pylori. PLoS Pathog. 2010;6: e1001026.](http://paperpile.com/b/1qmwpL/tV1zA)

3.	[Ling J, Cho C, Guo L-T, Aerni HR, Rinehart J, Söll D. Protein aggregation caused by aminoglycoside action is prevented by a hydrogen peroxide scavenger. Mol Cell. 2012;48: 713–722.](http://paperpile.com/b/1qmwpL/nYoPP)

4.	[Dwyer DJ, Belenky PA, Yang JH, MacDonald IC, Martell JD, Takahashi N, et al. Antibiotics induce redox-related physiological alterations as part of their lethality. Proc Natl Acad Sci U S A. 2014;111: E2100–9.](http://paperpile.com/b/1qmwpL/6wXTo)

5.	[Kwong SM, Ramsay JP, Jensen SO, Firth N. Replication of Staphylococcal Resistance Plasmids. Front Microbiol. 2017;8: 2279.](http://paperpile.com/b/1qmwpL/TdqmM)

6.	[Bertoni A, Folgieri R, Valentini G. Bio-molecular cancer prediction with random subspace ensembles of support vector machines. Neurocomputing. 2005;63: 535–539.](http://paperpile.com/b/1qmwpL/Z5Gc)
